# Pharmacological targeting of CBX7 alters the epigenetic landscape and induces differentiation of leukemic cells

**DOI:** 10.1016/j.bneo.2024.100052

**Published:** 2024-10-24

**Authors:** Anne P. de Groot, Chelsea R. Wilson, Ellen Weersing, Jacobine S. Pouw, Albertina Dethmers-Ausema, Huong Nguyen, Evan F. W. Chen, Alok Shaurya, Linda Smit, Fraser Hof, Gerald de Haan

**Affiliations:** 1 European Research Institute for the Biology of Ageing, University Medical Center Groningen, Groningen, The Netherlands; 2 Department of Hematopoiesis, Sanquin Research and Landsteiner Laboratory, Amsterdam UMC, Location University of Amsterdam, Amsterdam, The Netherlands; 3 Department of Chemistry, University of Victoria, Victoria, BC, Canada; 4 Centre for Advanced Materials and Related Technology, University of Victoria, Victoria, BC, Canada; 5 Department of Hematology, Amsterdam University Medical Center, Location Vrije Universiteit Medical Center, Cancer Center Amsterdam, Amsterdam, The Netherlands

## Abstract

Self-renewal of leukemic cells results in the accumulation of dysfunctional blood cells and suppression of normal hematopoiesis. The polycomb group protein chromobox 7 (CBX7) is an epigenetic regulator that represses genes required for differentiation and cell cycle arrest and thereby promotes self-renewal. Because leukemic cells are highly self-renewing, we tested whether pharmacological targeting of CBX7 would reduce self-renewal and induce differentiation of human leukemic cells. We found that existing and newly developed CBX7 inhibitors derepress the epigenome, resulting in reduced ubiquitination of histone 2A and reduced binding of CBX7 to its target genes. This led to reduced cell growth, increased differentiation of leukemic cells in vitro, and delayed engraftment of primary leukemic cells in xenotransplant models. Therefore, pharmacological targeting of CBX7 constitutes a novel therapeutic approach for leukemia.

## Key Points

• CBX7 is essential for leukemic cell survival.
• Small-molecule inhibitors of CBX7 epigenetically reprogram leukemic cells and induce differentiation.

## Visual Abstract

**Figure:**
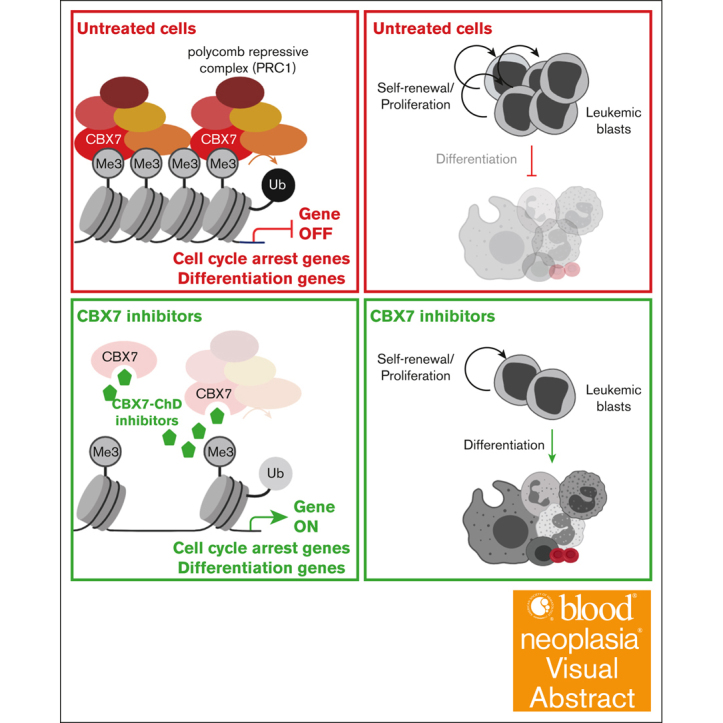

## Introduction

Hematopoietic stem and progenitor cells (HSPCs) can self-renew and differentiate toward mature blood cells, thus ensuring blood production throughout life.^1^ Epigenetic regulators dictate fate decisions that affect self-renewal and differentiation of HSPCs in a reversible and dynamic manner. Genes encoding proteins involved in differentiation are epigenetically repressed in HSPCs, whereas upon differentiation these primitive cells transform to an epigenetic state in which genes that regulate self-renewal are repressed. The balance between self-renewal and differentiation is strictly controlled to avoid the onset of leukemia.^2^

In leukemic cells, this balance is skewed toward self-renewal, resulting in an accumulation of immature blast cells in the blood and bone marrow (BM) accompanied by suppression of mature blood cell lineages. This results in anemia, bleeding, and susceptibility to infections. The prognosis for patients with leukemia remains poor,^3^^,^^4^ mainly because of leukemic cells that escape therapy, followed by relapse of the disease.^5^ In leukemic cells, enhanced self-renewal and impaired differentiation partially emerge from a dysregulated epigenetic landscape. DNA methylation profiles are aberrant and histone modification patterns are altered because of mutations in epigenetic regulators, such as the DNA methylation–associated proteins DNA (cytosine-5)-methyltransferase 3A (DNMT3A), isocitrate dehydrogenase 1/2 (IDH1/2), and Ten-Eleven Translocation-2 (TET2); and the chromatin modifiers Addition Sex Combs-like 1 (ASXL1), lysine methyltransferase 2A (KMT2A), and enhancer of zeste 2 polycomb repressive complex 2 subunit (EZH2).^6^

Although leukemia therapy mainly consists of nonselective chemotherapy, optionally followed by BM transplantation,^7^ multiple approved therapies targeting recurrently mutated epigenetic proteins with DNA demethylation agents, or IDH inhibitors have been reported as effective treatment strategies for leukemia.^6^^,^^8^ These studies highlight the potential of therapeutically targeting epigenetic proteins to reverse the epigenetic landscape and thereby restore the balance between self-renewal and differentiation in leukemic cells. Promising preclinical and clinical studies include therapeutically targeting polycomb group (PcG) proteins,^9^ which are key regulators of HSPC self-renewal.^10^, ^11^, ^12^, ^13^, ^14^, ^15^, ^16^

In HSPCs, PcG proteins form multimeric complexes that bind and modify histones, resulting in epigenetic silencing of genes important for cell cycle arrest and differentiation. Mutated or aberrant expression of PcG proteins in HSPCs is associated with leukemogenesis.^12^^,^^17^ The 2 best characterized PcG complexes are the polycomb repressive complex 1 (PRC1) and 2 (PRC2). The PRC2 subunit EZH2 catalyzes trimethylation of lysine 27 on histone 3 (H3K27me3), which is recognized by PRC1 via its chromobox (CBX) subunit (either CBX2, CBX4, CBX6, CBX7, or CBX8) leading to ubiquitination of lysine 119 on histone 2A (H2Aub119) followed by chromatin compaction and gene silencing.^18^

We have previously shown that among all CBX subunits, CBX7 is preferentially important to keep HSPCs in a stem cell state. CBX7 expression is high in HSPCs and decreases once cells start to differentiate.^14^ CBX7 differs from the other CBX proteins because it lacks the compaction and phase separation domain necessary for lineage commitment.^19^^,^^20^ Moreover, enforced overexpression of CBX7 but not of its close homologs CBX2, CBX4, or CBX 8 promotes HSPC self-renewal.^14^^,^^15^

Eventually, enforced overexpression of CBX7 in HSPCs results in leukemogenesis.^14^^,^^21^^,^^22^ Moreover, CBX7 can interact with mutant DNMT3A, and aberrant recruitment of PRC1 to differentiation-associated genes in DNMT3A-mutated HSCs results in a differentiation block and leukemic transformation.^23^ Downregulation of CBX7 expression in leukemic cells results in blocked proliferation and induction of differentiation.^15^

Thus, CBX7 is an important regulator for balancing self-renewal and differentiation in HSPCs and could be a potential therapeutic target to overcome the block of differentiation and excessive self-renewal of leukemic stem and progenitor cells. Multiple pharmacological compounds targeting the PcG proteins are under development and are studied in cancer biology,^9^ of which the CBX7-inhibitor MS452 has been demonstrated to block binding of CBX7 to the INK4A/ARF locus and thereby derepress cell cycle arrest genes in prostate cancer.^9^^,^^24^

In this study, we explored whether existing and newly synthesized CBX7 inhibitors could epigenetically reprogram leukemic cells to inhibit proliferation and induce differentiation. We found that pharmacological inhibition of CBX7 reduced levels of H2A ubiquitination (H2Aub) and released CBX7 binding to target loci that encode genes that regulate differentiation and cell cycle arrest. These molecular events reduced proliferation and induced differentiation of leukemic cell lines and primary patient samples. Most notably, a short ex vivo exposure of primary leukemic cells to CBX7 inhibitors reduced engraftment of leukemia-initiating cells in immunodeficient mice.

## Methods

### Cell lines and human primary samples

The leukemic cell lines OCI-AML3, KG-1, EOL-1, MUTZ8, SupB15, Nalm-6, and REH were cultured in RPMI 1640 media. Primary acute myeloid leukemia (AML) and cord blood (CB) samples were cultured in StemSpan serum-free expansion medium or α-modification Eagle minimum essential medium. Informed consent was obtained per Declaration of Helsinki. All cells were maintained at 37°C, in a humidified atmosphere containing 5% CO_2_.

### Mice

NOD.Cg-Prkdc^Scid^Il2rg^tmlWjl^/SzJ (NSG) mice were bred and maintained under defined conditions at the central animal facility within the University Medical Center Groningen. All experiments were approved by the central commission for animal testing and animal ethical committee.

### Generation of CBX7^+/−^ OCI-AML3 cell lines

CRISPR-associated protein 9 (Cas9) ribonucleoprotein (RNP) complexes were made by incubating Cas9 with duplexed crRNA4 single-guide RNA (sgRNA) and nucleofection into OCI-AML3 cells. After nucleofection, cells were single-cell sorted to generate single-cell–derived clones that were subsequently screened for their genotype.

### In vitro growth experiments with CBX7^+/−^ OCI-AML3 cells

At day 0, 250 000 CBX7^+/−^ or wild-type CBX7 (CBX7^wt^) OCI-AML3 cells per mL were cultured in RPMI 1640. After 4 days of culture, cells were counted manually to assess viability.

### EC compound synthesis

Detailed EC compound synthesis can be found in supplemental Methods and the full synthetic scheme in supplemental Figure 2A.

### Treatment with CBX7 or EHMT1/2 inhibitors in leukemic cell lines and downstream effects

Leukemic cells were treated with increasing concentrations of the CBX7 inhibitors MS452 (Sigma, SML1405), EC-134 (synthesized in this study), or BDA-41 (gift from Zhang^25^), or with increasing concentrations of the EHMT1/2 inhibitor UNC0642 (Sigma, SML1037) for 4 days. Flow cytometric analysis was performed to measure the effect of the inhibitors on histone modifications, cell cycle, proliferation rate, differentiation, and apoptosis. Chromatin immunoprecipitation experiments were performed to check for CBX binding to chromatin after treatment.

### Xenotransplantation of ex vivo CBX7-inhibitor–treated AML cells

Primary AML cells were treated with MS452 or BDA-41. After 24 hours, cells were harvested and transplanted into irradiated NSG mice. After transplantation, every 2 or 3 weeks, peripheral blood (PB) was obtained to determine the human CD45^+^ (hCD45^+^) leukemic cell engraftment levels over time, using flow cytometry. At the end of the experiment, the BM and spleen were removed to determine engraftment of leukemic cells in these organs.

### Quantification and statistical analysis

Data were plotted into graphs using GraphPad Prism. All flow data were analyzed by FlowJo software (BD).

Statistical details for each experiment are indicated in the legend of each figure, as appropriate.

Detailed information about the methods is described in supplemental Methods.

## Results

### CBX7 is essential for leukemic cell survival

Because CBX7 is an important regulator of self-renewal in normal HSPCs and because leukemic cells have enhanced self-renewal activity, we asked whether blocking the activity of CBX7 would impair leukemic cell growth. We used multiple independent strategies to inhibit CBX7 in leukemic cells.

**Figure 1.**
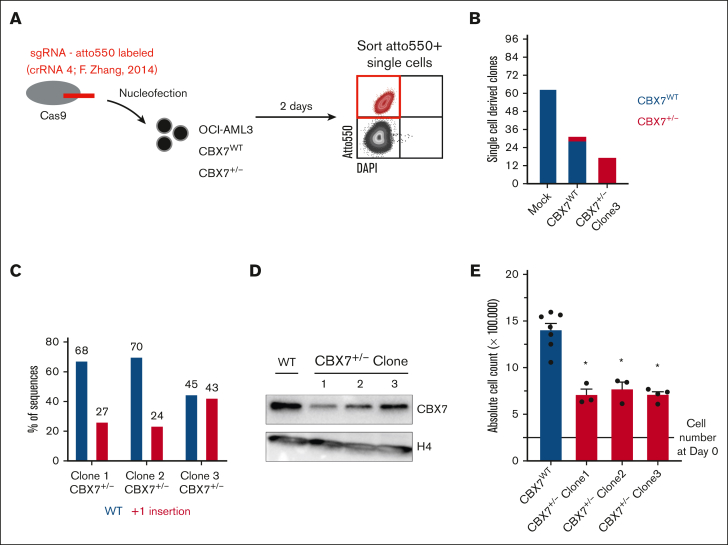
**CBX7 gene targeting using CRISPR-Cas9 results in the outgrowth of WT or heterozygous hit but not homozygous hit OCI-AML3 clones.** (A) Experimental setup. RNP complexes (Cas9 plus an atto550-labeled sgRNA targeting CBX7 validated in F. Zhang’s knockout library screen) are transfected into OCI-AML3-CBX7^WT^ or already heterozygous hit OCI-AML3-CBX7^+/−^ cells, by nucleofection. After 2 days of recovery, Atto550^+^ cells are single-cell sorted into 96 wells. (B) Amount of Atto550^+^ sorted single-cell–derived clones after nucleofection of mock or CBX7-targeting sgRNAs in either CBX7^WT^ or CBX7^+/−^ OCI-AML3 cells. Bars indicate the number of single-cell–derived clones, with blue indicating the amount of outgrown WT CBX7 clones and red the outgrown of heterozygous hit CBX7 clones. (C) Percentage of polymerase chain reaction (PCR)–amplified WT CBX7 sequence, or insertion-mutated CBX7 sequence in the 3 heterozygous hit OCI-AML3 clones. (D) Western blot analysis showing CBX7 and H4 protein expression levels in the 3 heterozygous hit OCI-AML3 clones. (E) Absolute cell count after 4 days of in vitro culturing 250 000 CBX7^WT^ or CBX7^+/−^ OCI-AML3 cells. Bars represent the mean of ≥3 replicates with standard error of the mean (SEM) as error bar. Student *t* test was used to calculate the *P* value between CBX7^WT^ and CBX7^+/−^ OCI-AML3 cells, ∗*P* < .05. DAPI, 4′,6-diamidino-2-phenylindole.

Previously, we showed that downregulation of CBX7 in AML cell lines using a lentiviral short-hairpin strategy reduces leukemic cell growth.^15^ Here, we investigated whether genetic deletion of CBX7 using a CRISPR/Cas9 approach would block leukemic cell proliferation. To do so, Cas9 and an atto550-labeled sgRNA targeting CBX7 were delivered as an RNP complex into OCI-AML3 cells. Transfected atto550^+^ OCI-AML3 cells were single-cell sorted (Figure 1A). In mock-transfected conditions we were able to isolate 62 clones from 96 single cells. OCI-AML3 cells that were exposed to the CBX7-targeting sgRNA resulted in fewer single-cell–derived clones (31/96). Three of these clones were heterozygous hits, and in no clones were both alleles altered by the RNP complex, suggesting that CBX7^−/−^ OCI-AML3 cells do not survive.

Retargeting the remaining CBX7 locus by delivering the RNP complex to 1 of the CBX7^+/−^ clones, led to even fewer single-cell–derived clones (17/96), of which none had a homozygous alteration (Figure 1B). All 3 CBX7^+/−^ clones had a +1 insertion at the Cas9 cut site, which results in a frameshift that codes for a truncated CBX7 protein (Figure 1C; supplemental Figure 1A-B). Furthermore, deleting 1 CBX7 allele resulted in downregulation of CBX7 protein levels in all 3 CBX7^+/−^ clones (Figure 1D). Similar to shCBX7,^15^ downregulation of CBX7 led to reduced leukemic cell growth (Figure 1E).

These data suggest that leukemic cells are not viable when lacking CBX7 and that reduced levels of CBX7 impair cell growth.

Our aim was to translate these findings to a more therapeutically relevant approach and assessed whether pharmacological targeting of CBX7 would have a similar effect to downregulating CBX7 expression in leukemic cells.

**Figure 2.**
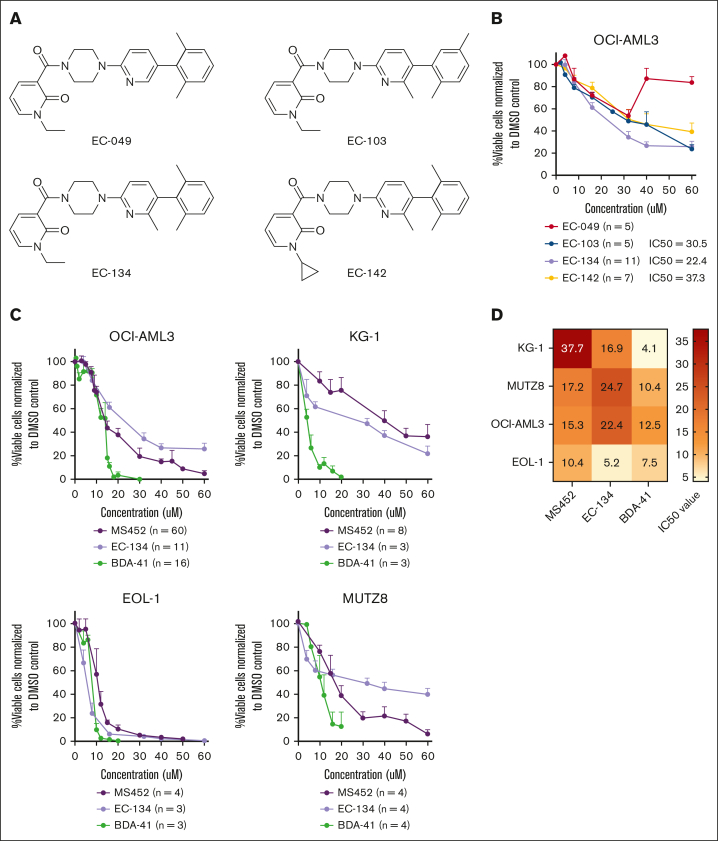
**CBX7 inhibitors reduce the viability of leukemic cell lines.** (A) Chemical structure of EC compounds synthesized for this study. (B) Percentage viable OCI-AML3 cells normalized to their experimental untreated DMSO control after treatment with the EC compounds for 4 days. Data points are plotted as the mean of ≥3 replicates with SEM as error bar. (C) Percentage viable cells normalized to their experimental untreated DMSO control of 4 myeloid leukemia cell lines after treatment with increasing doses of the CBX7 inhibitors, MS452, EC-134, and BDA-41 for 4 days. Data points are plotted as the mean of ≥3 replicates with SEM as error bar. (D) IC_50_ values of each compound in the different cell lines are indicated in the heat map.

First, we explored the potency of newly synthesized small-molecule CBX7 inhibitors. A high-throughput screen of AstraZeneca compound libraries was conducted to identify new lead structures for CBX7 inhibition, in which the primary readout was by fluorescence polarization.^26^ A new structural class of CBX7 inhibitors based on a substituted N-alkyl pyridone was identified. A small library of synthetic derivatives was prepared (supplemental Figure 2A-B), leading to 4 small molecules with the ability to disrupt the CBX7 chromodomain–histone tail interaction (50% inhibitory concentration [IC_5__0_] < 60 μM; Figure 2A; supplemental Figure 2C). Preliminary studies of cell-based activities led to the discovery of compound EC-134 as a low–molecular weight, cell-permeable, and most potent (IC_50_ = 22.4 μM) EC compound to reduce leukemic cell viability (Figure 2A-B).

Thereafter, we exposed multiple AML or acute lymphoid leukemia (ALL) cell lines to the newly synthesized inhibitor EC-134, as well as to the CBX7 inhibitors MS452 and BDA-41 (see Table 1 for information about the inhibitors). A dose-dependent reduction in leukemic cell viability was observed with all 3 compounds in the AML cell lines OCI-AML3, KG1, EOL1, and MUTZ8 (Figure 2C-D) and for 2 of the inhibitors in the acute B-cell lymphoid leukemic (B-ALL) cell lines Nalm-6, Sup-B15, and REH (supplemental Figure 3). Overall, ALL cells showed higher sensitivity to CBX7 inhibitors than AML cells. MS452 and EC-134 showed similar dose-dependent kinetics in reducing leukemic cell viability, whereas BDA-41 showed very distinct kinetics but was the most potent compound to reduce leukemic cell viability (Figure 2C-D). Collectively, these genetic and pharmacological data support earlier short-hairpin RNA studies and reveal that leukemic cell survival depends on CBX7.

**Table 1.** **Overview of small molecules used in the study**

Small molecule	Target	Proposed mode of action	Evidence	Source
MS452	CBX7	Prevents binding of chromodomain to H3K27me3	In vitro	24,27
EC-134	CBX7	Disrupt CBX7 chromodomain–histone tail interaction		AstraZeneca compound library
BDA-41	CBX7	ATCase inhibitor, which interacts with CBX7	In silico	26
UNC0642	EHMT1 and EHMT2	Blocks catalytic domain of the H3K9 methyltransferases EHMT1 and EHMT2	In vitro, in vivo	28

### Pharmacological targeting of CBX7 alters the epigenetic landscape of leukemic cells

To understand the mechanism by which CBX7 inhibitors reduce leukemic cell viability, we examined whether these inhibitors affect the epigenetic landscape in leukemic cells. CBX7 regulates gene repression by binding to H3K27me3 and recruiting the PRC1 complex to target loci. This results in H2AKub119 and chromatin compaction. CBX7 inhibitors are considered H3K27me3-competitive inhibitors that prevent this repressive activity.

**Figure 3.**
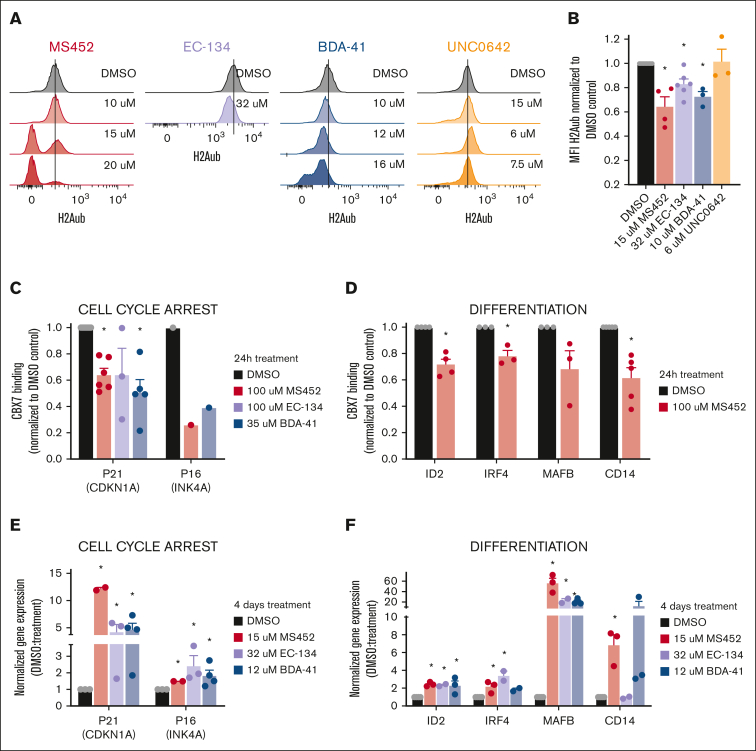
**CBX7 inhibitors change the epigenetic landscape of OCI-AML3 cells to an active state.** (A-B) H2Aub expression levels in OCI-AML3 cells after 4 days of in vitro treatment. (A) Representative histograms of the mean fluorescent intensity (MFI) of H2Aub-AF647 signal obtained with flow cytometry in untreated DMSO control cells (black), and after treatment with increasing concentrations of MS452 (red), EC-134 (purple), BDA-41 (blue), or UNC0642 (orange); see supplemental Figure 2A for complete gating strategy. (B) Quantification of the MFI of H2Aub-AF647 normalized to the DMSO control. Bars represent the mean of ≥3 replicates with SEM as error bar. Student *t* test was used to calculate the *P* value between DMSO and treatment, ∗*P* < .05. (C-D) CBX7 binding to the chromatin at (C) cell cycle arrest and (D) differentiation-associated target genes after 24 hours of treatment with MS452, EC-134, or BDA-41 in OCI-AML3 cells overexpressing the fusion protein CBX7-GFP. Chromatin immunoprecipitation quantitative PCR (qPCR) experiments were performed to calculate percent input, which was used to normalize the CBX7 binding in the treated samples to their experimental DMSO control; see supplemental Table 1 for raw data. Bars represent the mean of ≥1 replicates with SEM as error bar. Student *t* test was used to calculate the *P* value between DMSO and treatment, ∗*P* < .05. (E-F) Upregulation of the (E) cell cycle arrest and (F) differentiation-associated target genes in OCI-AML3 cells after 4 days of treatment with MS452, EC-134, or BDA-41. CDKN1A, INK4A, ID2, IRF4, MAFB, and CD14 relative to HPRT messenger RNA (mRNA) expression were obtained with qPCR and normalized to their experimental DMSO control (2^−ΔΔCt^). Bars represent the mean of ≥2 replicates with SEM as error bar. Student *t* test was used to calculate the *P* value between DMSO and treatment, ∗*P* < .05.

As expected, we observed that treatment of OCI-AML3 cells with the CBX7 inhibitors led to a dose-dependent global reduction of H2Aub levels (Figure 3A). Around their respective IC_50_ concentration values H2Aub levels were shown to be significantly different from the dimethyl sulfoxide (DMSO) control (Figure 3B), suggesting that the CBX7 inhibitors prevent PRC1-dependent gene repression. Reduction of H2Aub levels was specific for the CBX7 inhibitors, because reduced viability induced by pharmacological targeting of other epigenetics regulators, such as H3K9 methyltransferases with UNC0642, did not affect ubiquitination of H2A whereas it reduced H3K9me2 levels (Figure 3A-B; supplemental Figure 4B-D).

Our previous work demonstrated that CBX7 targets and represses cell cycle arrest and differentiation-associated genes in HSPCs^15^ (supplemental Figure 4E-F). To test whether CBX7 inhibitors derepressed those known target genes, such as p21, p16, ID2, IRF4, MAFB, and CD14, we assessed CBX7 occupancy at these loci by chromatin immunoprecipitation quantitative polymerase chain reaction, after exposure of OCI-AML3 cells to the CBX7 inhibitors. We observed that treatment with CBX7 inhibitors indeed led to reduced binding of CBX7 to those target genes (Figure 3C-D; supplemental Table 1). Loss of CBX7 binding to target loci was accompanied by increased expression of these transcripts (Figure 3E-F). Although the distinct kinetics in Figure 2C suggest that these molecules may also target other proteins, the CBX7 inhibitors preferentially blocked recruitment of CBX7 (but not CBX2, CBX4, CBX6, or CBX8) to the p21 locus (supplemental Figure 4G).

In conclusion, these data show that CBX7 inhibitors induce a global switch from a repressive landscape toward a more active epigenetic landscape, whereby genes important for cell cycle arrest and differentiation become accessible for transcription.

### CBX7-inhibitors block proliferation, induce terminal differentiation, and promote apoptosis of leukemic cells

**Figure 4.**
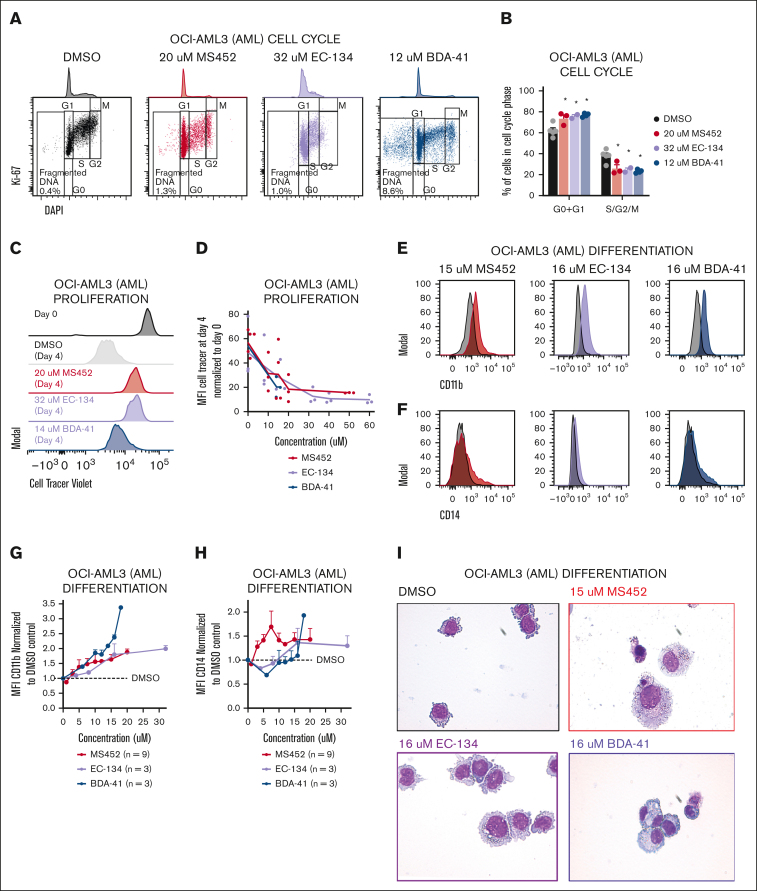

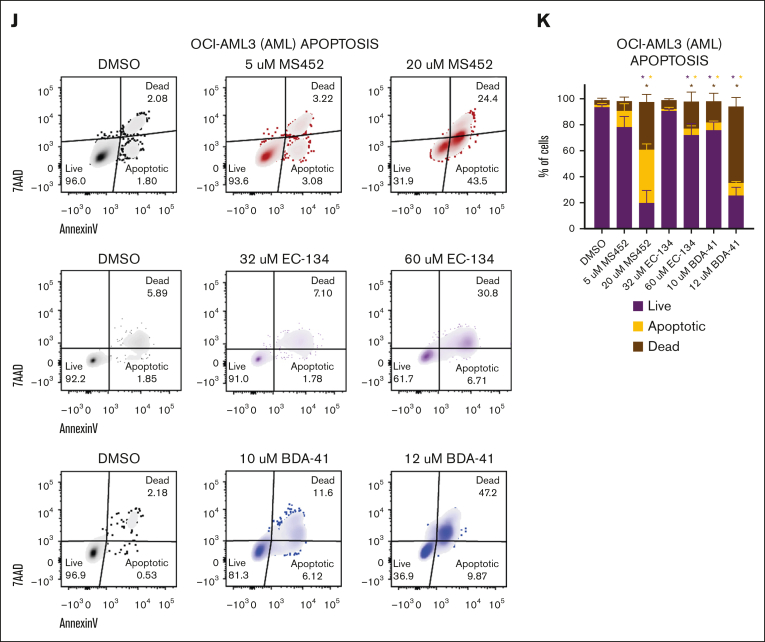
**CBX7 inhibitors block proliferation, induce terminal differentiation, and promote apoptosis of OCI-AML3 cells.** Phenotype of OCI-AML3 cells after treatment with MS452, EC-134, or BDA-41 for 4 days. (A-B) Cell cycle analysis. (A) Representative flow plots of Ki67-AF488 and DAPI (4′,6-diamidino-2-phenylindole) signal indicating the cell cycle phases G0, G1, S, G2, and M, and fragmented DNA. (B) Quantification of the percentage of the unfragmented cells in G0 + G1 and S + G2 + M phase of ≥2 replicates with SEM as error bar. Student *t* test was used to calculate the *P* value between DMSO and treatment, ∗*P* < .05. (C-D) Proliferation rate. (C) Representative histograms of the MFI of the CellTrace Violet signal in cells at the start of treatment (black), and after 4 days without treatment (gray), or MS452 (red), EC-134 (purple), or BDA-41 (blue) treatment. (D) Quantification of the proliferation rate was calculated by dividing the CellTrace Violet MFI at day 4 from the MFI at day 0. Lines go through the means of the individual data points of ≥2 replicates. (E-I) Differentiation potential. (E-F) Representative histograms of the MFI of (E) CD11b-BV421 or (F) CD14-AF700 obtained with flow cytometry in untreated DMSO-treated control cells (black), and MS452- (red), EC-134– (purple) or BDA-41–treated (blue) cells. (G-H) Normalized expression of (G) CD11b and (H) CD14 calculated by normalizing the MFI of the treated samples to the MFI of their experimental DMSO control. Data points are plotted as the mean of ≥3 replicates with SEM as error bar. (I) May-Grünwald Giemsa staining of untreated DMSO control cells or cells treated with CBX7 inhibitors. (J-K) Apoptosis induction. (J) Representative flow plots of 7-aminoactinomycin D (7-AAD) and annexin-V–BV421 signal indicating the gates for live, apoptotic, or dead cell populations. (K) Quantification of the percentage of cells in each gate. Bars represent the mean of n = 8 for DMSO, n = 3 for MS452, n = 2 for EC-134, and n = 3 for BDA-41 with SEM as error bar. Student *t* test was used to calculate the *P* value between treatment and their experimental DMSO control, ∗*P* < .05, with green stars indicating differences in live cells, orange stars indicating differences apoptotic cells, and red stars indicating differences dead cells.

To investigate to what extent these epigenetic alterations affect leukemic cell behavior, we assessed proliferation and differentiation potential after treatment with the CBX7 inhibitors. We observed a reduced G1-S transition whereby cells accumulate in G1 phase (Figure 4A-B). To check how the block in the cell cycle affected leukemic cell division, we tracked the proliferation rate by labeling cells with a cell tracer proliferation dye. Whereas cells retained the dye when exposed to CBX7 inhibitors, untreated cells lost the dye over time, indicating reduced proliferation upon exposure to CBX7 inhibitors (Figure 4C-D). Instead, CBX7 inhibition of AML cells induced differentiation, as shown by increased expression of the differentiation markers CD11b (Figure 4E,G; supplemental Figure 5A-B) and CD14 (Figure 4F,H), of which CD11b is not expressed and CD14 is moderately expressed by undifferentiated OCI-AML3 cells. Moreover, we show morphological changes toward mature monocytes and macrophages (Figure 4I). Unexpectedly, because EC-134 did not derepress CD14 (Figure 3F), this compound, similar to the other CBX7 inhibitors, induced upregulation of CD14 at the protein levels (Figure 4F,H). Similar results were found in ALL cells, which maintained the proliferation dye and gained the lymphoid differentiation marker CD20, accompanied by reduction of the lymphoid precursor marker CD10 (supplemental Figure 5C-G).

A block in proliferation accompanied by induced differentiation only partially explains the reduced viability of leukemic cells that we observed in our cultures (Figure 2A). Therefore, we investigated whether the CBX7 inhibitors induced cell death. The increase in fragmented DNA (Figure 4A; supplemental Figure 5H) after CBX7 inhibitor exposure suggested that OCI-AML3 cells indeed undergo cell death. Furthermore, we observed that the majority of AML cells undergo apoptosis after treatment with CBX7 inhibitors, as shown by an excess of annexin-V–positive, 7-aminoactinomycin D–positive cells, instead of nonapoptotic cell death shown by a small number of annexin-V–positive, 7-aminoactinomycin D–positive cells after CBX7 inhibitor treatment (Figure 4J-K; supplemental Figure 5I-J). We conclude that CBX7 inhibitors induce leukemic cell death via apoptosis either directly of the leukemic blast cells or of their differentiated progeny.

Altogether, altering the epigenetic landscape by pharmacologically inhibiting CBX7 reactivates molecular pathways that result in cell cycle arrest, differentiation, and apoptosis, causing reduced leukemic cell survival.

### CBX7 inhibitors reduce the proliferation of primary leukemic blasts but not of normal HSPCs

**Figure 5.**
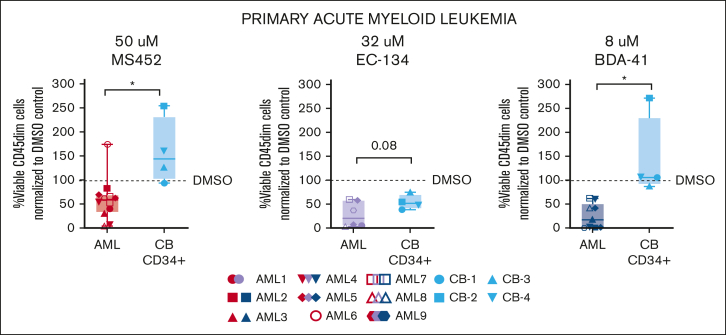
**Primary, patient-derived leukemic cells are sensitive to CBX7 inhibition whereas normal primitive CB-derived CD34^+^ cells are insensitive.** Percentage of viable immature (CD45dim) AML or CD34^+^ CB cells normalized to their experimental DMSO control after 1 week of in vitro treatment with MS452 (left panel), EC-134 (middle panel), or BDA-41 (right panel). Cells were cultured in StemSpan supplemented with SCF, TPO, and FLT3L. Box plots represent the median of ≥4 different AML or CB samples. Student *t* test was used to calculate the *P* value between the AML and CB samples, ∗*P* < .05.

To determine whether CBX7 inhibitors reduced the survival of primary AML cells we exposed multiple patient-derived AML samples (see supplemental Table 2 for patient information and supplemental Figure 6A for CBX7 expression levels) to the inhibitors MS452, EC-134, and BDA-41. To assess whether leukemic cells would be more sensitive compared with nonmalignant cells, we also exposed normal CD34^+^ CB HSPCs to these inhibitors. All 3 inhibitors similarly reduced the viability of AML blast cells (Figure 5). In contrast, MS452 and BDA-41 but not EC-134 did not reduce the viability of normal CD34^+^ CB cells after 1 week of treatment (Figure 5; supplemental Figure 6B). We did not observe strong effects on differentiation, other than a minor upregulation of CD45 and CD11b, and downregulation of CD34/117 expression in some AML cases (supplemental Figure 6B-F). To increase AML cell proliferation, we enriched the medium of AML cells and of normal CD34^+^ cultures with interleukin-3 and granulocyte colony-stimulating factor. Upon exposure of cells to MS452 or EC-134, cell growth of most AML samples (8/11) but less so of CD34^+^ CB samples (4/7) was reduced (supplemental Figure 6G). In agreement, AML cells were less viable in these cultures whereas CD34^+^ CB cell viability was only moderately affected (supplemental Figure 6H). Similar results were obtained with primary ALL cells (supplemental Figure 6I-J).

Thus, these data demonstrate that CBX7 inhibitors impair cell growth of patient-derived primary leukemic cells. We did not observe a correlation between cellular response toward CBX7 inhibition and mutational status of the cells. In contrast, healthy HSPCs were largely spared by this treatment strategy, revealing a therapeutic window to target leukemic cells.

### Ex vivo exposure of CBX7 inhibitors delays leukemia formation in vivo

Because we observed that CBX7 inhibitors impair leukemic cell survival and trigger those cells to differentiate, we wanted to investigate whether CBX7 inhibitors could delay the onset of leukemia in vivo. Of the different CBX7 inhibitors tested in this study, EC-134 showed lowest potency. This compound had the highest IC50 value, could not derepress all tested target genes, and had a much weaker effect on the reduction of H2Aub and on induction of apoptosis. Therefore, for in vivo experiments we decided to continue with MS452 and BDA-41.

**Figure 6.**
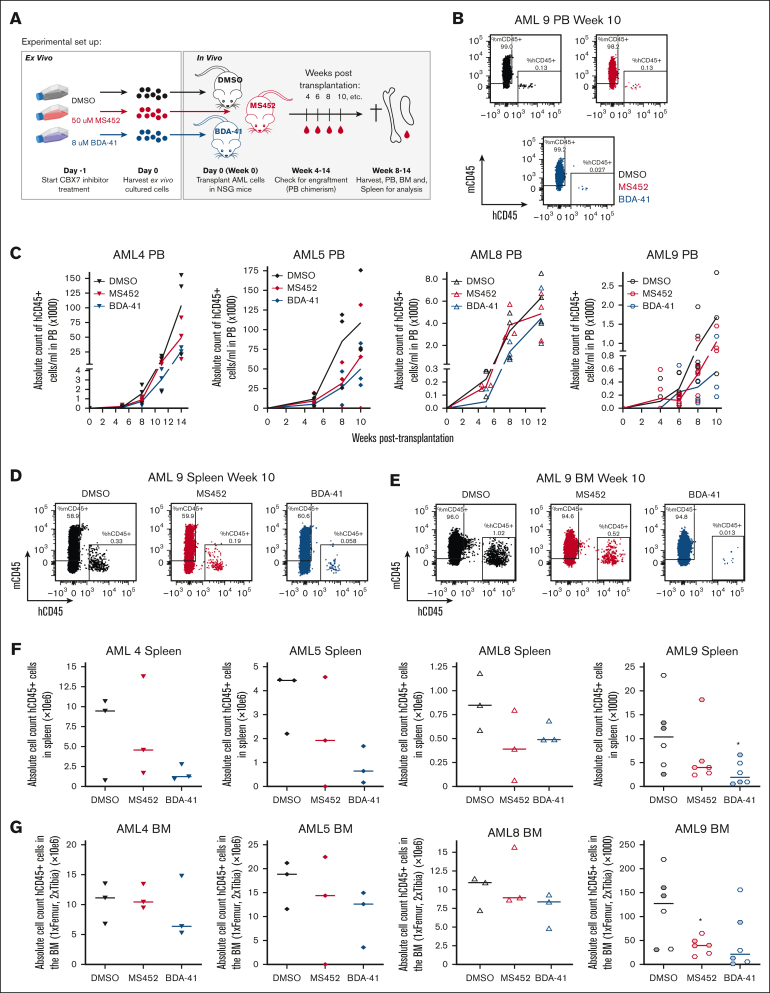
**CBX7 inhibitors delay leukemogenesis.** (A) Experimental setup. Primary AML cells were ex vivo culture and exposed to DMSO only, 50 μM MS452, or 8 μM BDA-41. After 24 hours, cells were harvested and equal number of cells per mouse were transplanted into irradiated immune-deficient mice. After transplantation, the PB was collected to check human AML cell chimerism in the mice over time. In the final week, mice were euthanized and the PB, BM, and spleen were collected and analyzed. (B-C) PB chimerism; (B) flow plots indicating the gates for mouse CD45^+^ cells and human AML CD45^+^ cell populations in the PB. (C) Amount of hCD45^+^ AML cells in the PB over time. Each symbol is a mouse. Lines go through the means of the individual data points of ≥3 mice. (D-G) BM and spleen analysis. (D-E) Flow plots indicating the gates for mouse CD45^+^ cells and human AML CD45^+^ cell populations in the (D) spleen and (E) BM. (F-G) Absolute human AML CD45^+^ cell count in the (F) spleen and (G) BM. Each symbol is a mouse, and medians are indicated as a flat line. For AML9, filled symbols indicate mice that were euthanized 8 weeks after transplantation, and open symbols indicate mice that were euthanized 10 weeks after transplantation. Student *t* test was used to calculate the *P* value between the DMSO- and CBX7-inhibitor– pretreated mice, ∗*P* < .05.

To do so, we exposed 4 different primary AML samples to the most potent antileukemic compounds MS452 and BDA-41. After 24 hours exposure with the CBX7 inhibitors ex vivo, we harvested cells and transplanted these into NSG mice. We did not observe reduced viability after 24 hours of treatment, and transplanted the same number of cells for each condition. During the engraftment period we measured human AML cell chimerism in the PB, and in the final week after transplantation we assessed human AML cell engraftment in the BM and infiltration in the spleen (Figure 6A). We observed a trend toward delayed engraftment of leukemic cells, with mice transplanted with pretreated CBX7-inhibitor cells showing slower kinetics of hCD45^+^ AML cells in the PB (Figure 6B-C). In addition, pretreatment with CBX7 inhibitors showed a trend toward reduced infiltration of hCD45^+^ AML cells in the spleen (Figure 6D,F) and reduced engraftment of hCD45^+^ AML cells in the BM (Figure 6E,G).

In conclusion, short-term exposure of leukemic cells to CBX7 inhibitors derepressed CBX7 target genes (Figure 3C-D) and reduced their in vivo engraftment potential (Figure 6).

## Discussion

In this study, we explored to what extent CBX7 is required for leukemic self-renewal and whether CBX7 inhibitors may be considered as antileukemia therapeutics.

In recent years, AML treatment has significantly improved because of the identification of recurrent mutations associated with molecular alterations in AML cells. This resulted in the US Food and Drug Administration approval of multiple drugs that interfere with these molecular alterations.^29^, ^30^, ^31^, ^32^, ^33^, ^34^, ^35^, ^36^, ^37^ However, nonselective chemotherapy, followed by HSPC transplantation, remains the main treatment option for patients without targetable mutations.^7^

In this study, we demonstrate that small molecules of different structural classes targeting CBX7 can epigenetically reprogram leukemic cells regardless of their mutational status. Because a dysregulated epigenetic landscape is a common hallmark of cancer, targeting key epigenetic regulators in leukemia has emerged as an attractive therapeutic approach. Clinical trials and approved epigenetic treatment strategies have been demonstrated to effectively target leukemic cells.^6^ These treatment strategies typically target recurrently mutated epigenetic proteins.^6^ Although CBX7 plays an important role in multiple cancers, either as an oncogene or tumor suppressor,^38^ CBX7 has been rarely found to be mutated in cancer. When browsing multiple leukemic data sets,^39^, ^40^, ^41^ we found only 2 CBX7 mutated cases: 1 patients with AML with 2 frameshift mutations (A66Gfs∗6 and G68Ifs∗4), and a patients with ALL with a stop-gain mutation (D64_R65insF∗). Indeed, our data suggest that CBX7 activity is essential for leukemic cells, regardless of their subtype, and, therefore, the antileukemic effect of CBX7 inhibition can most likely be translated to any immature hyperproliferative leukemia type.

The development of potent, selective, and cell-permeable CBX inhibitors has been difficult because of the molecular malleability of the chromodomain and the significant sequence similarity between the chromodomains of the various CBX proteins.^9^ Nevertheless, multiple CBX inhibitors have become available, such as inhibitors that preferentially target CBX2,^42^ CBX6,^43^ CBX7,^24^^,^^27^ and CBX8.^44^^,^^45^ High concentrations of the inhibitors are often required to have an effect in cells. We also explored the antileukemic effect of the dual-specific CBX4/7 inhibitors UNC3866^46^ and UNC4976^47^ but observed little to no effect on THP-1 and OCI-AML3 cells, with IC_50_ values ranging from 130 to 320 μM (data not shown). We found MS452^24^ as the best commercially available CBX7 inhibitor for leukemia treatment. Similar to the anticancer activity of MS452 with concentrations up to 250 μM in glioma^48^ and 500 μM in prostate cancer,^24^ we observed antileukemic effects using high MS452 concentrations up to 100 μM. We tested 2 novel CBX7 inhibitors of which EC-134 showed a similar dose-response range as MS452, and BDA-41 showed an improved potency with lower IC_50_ concentrations. However, the distinct dose-response kinetics between cell lines may suggest that targets other than CBX7 may be affected in certain cell lines. Notwithstanding potential off-target effects, we observed direct effects on CBX7 binding to chromatin and qualitatively very similar phenotypes after treatment with 3 unrelated inhibitors.

MS452, EC-134, and BDA-41 all blocked proliferation and induced differentiation of both AML and ALL cells, although ALL cells were more sensitive to the CBX7 inhibitors. This may be explained by the fact that ALL cells in general are more prone to apoptosis than AML cells.^49^^,^^50^

Differentiation therapy is a clinically effective approach for acute promyelocytic leukemia, but translating this strategy successfully to non–acute promyelocytic leukemia has remained difficult. Recently, it has been shown possible to overcome the differentiation block of leukemic cells by interfering with epigenetic proteins using IDH inhibitors, histone deacetylase inhibitors, KMT2A complex inhibitors, or using vitamin C to restore TET activity. However, leukemias harboring mutations in genes that are targeted by these compounds preferentially benefit from these treatments.^51^ Upregulation of the CBX7 target genes ID2,^52^, ^53^, ^54^ IRF4,^55^^,^^56^ and MAFB^57^, ^58^, ^59^, ^60^, ^61^, ^62^, ^63^, ^64^ stimulate myeloid differentiation ^52^^,^^53^^,^^55^, ^56^, ^57^^,^^59^ and overcome the differentiation block of leukemic cells,^54^^,^^58^^,^^60^, ^61^, ^62^ similar to CBX7 inhibitor treatment.

We also found that short-term exposure of leukemic cells to CBX7 inhibitors delayed leukemia formation in vivo. The observed in vivo effect is moderate, most likely because CBX7 is only temporarily and reversibly inhibited before leukemic cells are transplanted.

To effectively treat leukemia, it is important that leukemic cells but not healthy HSCs are selectively targeted and killed by the treatment strategy. Unexpectedly, because CBX7 is also important for normal HSCs,^14^^,^^15^ we found that CBX7 inhibitors preferentially target leukemic cells. Leukemic cells appear more dependent on CBX7 than normal HSPCs. This could be caused by (1) a distinct epigenome of normal and leukemic cells, (2) variant CBX-containing complexes that are more active in leukemic cells, or (3) upregulated CBX7 expression in leukemic cells.

First, compared with normal HSPCs, H3K27me3 levels are reduced in leukemic cells.^65^ Because CBX7 inhibitors antagonize the recognition of H3K27me3 by CBX7, low levels of H3K27me3 in leukemic cells may explain a more rapid response and higher sensitivity of leukemic cells. Moreover, the PRC1 subunits BMI1 and RING1 that ubiquitinate H2AK119 are upregulated in leukemia^66^^,^^67^ and H2Aub levels maintain leukemic cells in an undifferentiated state.^68^ Thus, leukemic cells may show increased sensitivity toward the CBX7 inhibitors. Similar to CBX7 inhibitor treatment, reduced levels of H2Aub induce differentiation of leukemic cells^69^^,^^70^ and BMI-1 inhibitors that reduce H2Aub levels also induce apoptosis and differentiation, resulting in reduced leukemic cell growth while sparing normal hematopoietic cells.^71^^,^^72^

Secondly, variant PRC1 complexes are known to regulate hematopoiesis and leukemogenesis.^73^ Alternative CBX-containing epigenetic complexes, such as CBX8-rearranged KMT2A^74^ and CBX8-BCOR,^75^^,^^76^ can promote leukemogenesis. Therefore, the therapeutic window of CBX7 inhibitors may result from CBX7 supporting leukemogenesis in a PRC1-independent way. Previously we have shown that CBX7 can interact with the epigenetic H3K9 methyltransferases SETDB1 and EHMT1/2 in leukemic cells, suggesting that CBX7 can regulate malignant self-renewal via regulating H3K9me3-regulated gene repression.^15^ In CRISPR dropout screens these H3K9 methyltransferases have been identified as genetic vulnerabilities in leukemia,^77^^,^^78^ and therapeutically targeting H3K9 methyltransferases is an effective antileukemia approach (this study, and as reported in previous studies^79^, ^80^, ^81^, ^82^, ^83^). Although we did not observe differential expression of the SETDB1 and EHMT1/2 between normal HSPCs and leukemic cells, we did observe that therapeutic targeting of these proteins does not affect normal HSPCs (data not shown). Similar to H3K27me3 levels, H3K9me3 levels are downregulated in leukemic cells compared with normal HSPCs,^84^ supporting the idea that demethylation of H3K9 via a noncanonical role of CBX7 contributes to the higher CBX7 inhibitor sensitivity of leukemic cells vs normal HSPCs.

Finally, differential expression of CBX7 between leukemic and normal HSPCs could explain the therapeutic window of CBX7 inhibitors. Although there is no strong evidence of CBX7 overexpression in leukemia, we do show that leukemic cells have higher CBX7 transcript levels than normal HSPCs.

Overall, our data support the notion that CBX7 regulates the balance between self-renewal and differentiation in leukemic cells. Pharmacologically targeting CBX7 represses self-renewal and induces differentiation. Therefore, CBX7 may constitute a novel therapeutic approach in leukemia.

Conflict-of-interest disclosure: The authors declare no competing financial interests.

## Supplementary Material

Supplemental Methods, Figures, Tables 2, 3, and References
