# Supplementary material for: Pharmacological targeting of CBX7 alters the epigenetic landscape and induces differentiation of leukemic cells
Source: Blood Neoplasia. 2024 Oct 24;1(4):100052. doi: 10.1016/j.bneo.2024.100052 (PMC12182855; doi:10.1016/j.bneo.2024.100052)
Supplement: Supplemental Methods, Figures, Tables 2, 3, and References [file BNEO_NEO-2024-000381-mmc1.pdf]

This supplementary file includes:

- Supplementary Material and Methods
- Figs. S1 to S6
- Tables S1 to S3
- References

SUPPLEMENTARY METHODS

Cell lines

The leukemic OCI-AML3 (DSMZ, ACC-582, RRID:CVCL_1844), KG-1 (ATCC, CCL-246, RRID:CVCL_0374), EOL-1 (DSMZ, ACC-386, RRID:CVCL_0258), SupB15 (ATCC, CRL-1929, RRID:CVCL_0103), Nalm-6, and REH (gift from J.J. Schuringa) cells, were cultured in RPMI 1640 (Gibco, 21875-091) supplemented with 1% Penicillin Streptomycin (Gibco, 15140-122), and 10% heat-inactivated fetal calf serum (hi-FCS) (Gibco, 10270-106). The leukemic MUTZ8 cells (DSMZ, ACC-689, RRID:CVCL_2130) were cultured in RPMI 1640 supplemented with 1% Penicillin Streptomycin, 10% hi-FCS (Gibco, 10270-106), and 10 ng/mL human recombinant (hr-) GM-CSF (PeproTech, 300-03).
The retroviral packaging cell line platinum Amphotropic (plat-A) (Cell Biolabs, RV-102, RRID:CVCL_B489) was cultured in DMEM (Gibco, 41966052) medium supplemented with 1% Penicillin Streptomycin, 1 ug/mL puromycin (Invivogen, #ant-pr), 10 ug/mL blasticidin (Invivogen, #ant-bl) and 10% hi-FCS (Gibco, 10270-106). All cell lines were maintained at 37°C, in a humidified atmosphere containing 5% CO2.

Human patient-derived leukemia samples

Primary AML cells (AML1-5, and 8-13) were obtained from AML patients hospitalized at Amsterdam UMC, location VUmc, the Netherlands, according to HOVON AML protocols and were provided by Dr. L Smit. Additional primary AML cells (AML6-7, and 14-16) were provided by Prof. Dr. JJ Schuringa from the Department of Experimental Hematology, University Medical Center Groningen (UMCG), the Netherlands after informed consent. Primary ALL cells were provided by Prof. Dr. ESJM de Bont and Dr. ME Belderbos from the Department of Pediatric Oncology and Hematology/Pediatrics, UMCG, the Netherlands after informed consent. The mutational and cytogenetic status of the patient samples are available in Table S2. Frozen primary samples were thawed in warm 20% hi-FCS (Gibco,10099-141) in PBS (Gibco, 14190-169), washed (100g for 10 minutes), and resuspended in 20% hi-FCS in PBS supplemented with 100 ug/mL DNAse (Roche, 11284932001), and 10 mM MgCl2. After 20 minutes of incubation at 37°C, cells were washed again (100g for 10 minutes) whereafter they were resuspended in their appropriate culture medium. Primary AML cells were cultured in either short-term culture medium

namely, StemSpan SFEM (Stemcell technologies, #09650) medium supplemented with 1% Penicillin Streptomycin, 50 ng/mL hr-TPO (R&D systems, #288-TP), 50 ng/mL hr-SCF (R&D systems, 255-SC), and 50 ng/mL hr-FLT3-Ligand (R&D systems, #308-FK) or in long-term culture medium namely, αMEM Eagle (Lonza, BE02-002F) medium supplemented with 1% Penicillin Streptomycin, 12.5% hi-FCS (Gibco,10099-141), 12.5% Horse Serum (Stemcell technologies, #06850), 1 μM hydrocortisone (Stemcell technologies, #74142), 57.2 μM β-mercaptoethanol, 20 ng/ml hr-TPO, 20 ng/ml hr-IL-3 (R&D systems, #203-IL), and 20ng/mL hr-G-CSF (R&D systems, #214-CS), at 37°C, in a humidified atmosphere containing 5% CO_2. Primary ALL cells were maintained in StemSpan medium supplemented with 1% Penicillin Streptomycin, 50 ng/mL hr-TPO, 50 ng/mL hr-SCF, 50 ng/mL hr-FLT3-Ligand, and 20 ng/mL hr-IL-7 (Preprotech, #200-07) at 37°C, in a humidified atmosphere containing 5% CO_2, for both short- and long-term experiments.

Human cord blood samples

Cord blood (CB) was obtained from healthy full-term pregnancies after informed consent by the Declaration of Helsinki from the Obstetrics Department at the Isala Hospital in Zwolle, the Netherlands.

To isolate CD34+ cells, cord blood was diluted 1:1 with PBS supplemented with 2mM EDTA and 0.2% BSA (Gibco, 15260037). Two units of diluted CB were layered on 1 unit of lymphoprep (Stemcell technologies, #07851) in a SepMate tube (Stemcell technologies, #85450) and centrifugated for 20 minutes at 800g without brakes. After centrifugation, the middle layer containing the mononuclear cells was harvested and washed twice with 2mM EDTA-, 0.2% BSA-PBS (800g for 5 minutes, followed by 200g for 10 minutes) after which, max. 100 *106 mononuclear cells were resuspended in 300 uL cold 2mM EDTA-, 0.2% BSA-PBS and incubated with 100 uL magnetic CD34 microbeads and 100 uL FCR blocking reagent (Miltenyi Biotec, 130-046-702) for 30 minutes at 4°C. To elute the CD34+ cells, cells were washed with 2mM EDTA-, 0.2% BSA-PBS (450g for 5 minutes) whereafter the cells were applied to an LS column (Miltenyi Biotec, 130-042-401/7) so that CD34- cells can flow through followed by firmly pushing the plunger to elute the CD34+ cells. CD34+ CB cells were frozen in Cryostor CS10 (Sigma, C2874) until needed for further experiments. For culture, CD34+ CB cells were cultured in the same medium as used for the primary cells they were compared to.

Mice

NOD.Cg-Prkdc^{Scid}Il2rg^{tmlWjl}/SzJ (NSG) (Cat# 005557, RRID:IMSR_JAX:005557) mice were bred and maintained under defined conditions at the central animal facility within the University Medical Center Groningen, meaning: individually ventilated cages with a max. of five animals per cage, 12-h light:dark cycle, sterilized bedding, and ad libitum sterile food, and water. Two days prior to transplantation, the drinking water of 6 to 16-week-old female NSG mice was supplemented with 100 mg/L Ciprofloxacin (Hikma, RVG 29032) to prevent infections, followed by sub-lethally irradiation with 1 Gy one day prior to transplantation. Two weeks after irradiation, the Ciprofloxacin was removed from the drinking water. All experiments were approved by the Central Commission for animal Testing and Animal Ethical Committee.

Generation of CBX7$^{+/-}$ OCI-AML3 cell lines

The sgRNA sequence (5’ – TCTTCCTATACCCCGATGCT – ‘3) for crRNA4 was obtained from the F. Zhang CRISPR Library (1) and was synthesized by “Integrated DNA Technologies, BV (IDT)”, Belgium. For the mock sgRNA, Alt-R CRISPR-Cas9 Negative Control crRNA#1 (#1072544, IDT) was used. To duplex the sgRNA with a tracrRNA, the sgRNA was mixed with ATTO550-labeled Alt-R tracrRNA (IDT, #1072532), both in a final concentration of 80 μM, and incubated at 95°C for 5 minutes followed by cooling down to room temperature (RT). Cas9 Ribonucleoprotein (RNP) complexes were made by incubating the Alt-R S.p. HiFi Cas9 protein (#1081060, IDT) with the duplexed sgRNA at a molar ratio of 1:2 for 20 minutes at RT. The RNP complexes were nucleofected into OCI-AML3 cells using the SF Cell Line 4D-Nucleofector X kit (Lonza, V4XC-2024) with the following conditions: 1*106 cells, 100 μL format cuvettes, program DS-138 on a 4D nucleofector X unit (AAF-1003X, Lonza). Following nucleofection, cells were cultured for 2 days. To generate single-cell derived clones, ATTO550+ cells were single-cell sorted using the 100 μm nozzle on the Sony SH800S cell sorter (Sony). Genotype of the single-cell derived clones was screened by isolating gDNA using the QIAamp DNA Micro Kit (56304, Qiagen) and PCR amplified with primers covering the cut side (see table S3).

Western blot

Protein extracts were obtained using RIPA buffer (ThermoFisher, 89900) according to manufacturing instructions. A 25G needle was used to ensure nuclear lysis. Protein extracts were 4x diluted with Laemmli Sample buffer (Bio-Rad, #1610747) supplemented with β-mercaptoethanol. Samples were denatured by boiling the mixture at 95°C for 10 minutes before the samples were submitted to electrophoresis in a precast polyacrylamide gel (Bio-Rad, #4561094-6). After electrophoresis, proteins were transferred to a nitrocellulose membrane according to the Trans-Blot Turbo Transfer kit (Bio-Rad, #1704270) instructions. Membranes were incubated with the primary antibodies, rabbit-anti-CBX7 (1:1000) (Abcam, ab21873, RRID:AB_726005), or mouse-anti-H4 (1:1000) (Active motif, #61521, RRID:AB_2793667) overnight at 4°C followed by secondary antibody incubation with HRP-conjugated anti-rabbit IgG (1:1000) (Cytvia, NA934, RRID:AB_772206) or anti-mouse IgG (1:1000) (Cytvia, NA931, RRID:AB_772210) for 1 hour at RT. After antibody incubation, proteins were visualized with the SuperSignal Femto substrate kit (ThermoFisher, #34095) using the ChemicDoc XRS+ imaging system (BioRad).

In vitro growth experiments with CBX7$^{+/-}$ OCI-AML3 cells

At day zero, 250 000 CBX7$^{+/-}$ or CBX7wt OCI-AML3 cells/mL were cultured in their standard RPMI culture medium. After four days of culturing, cells were counted manually using a hemocytometer to assess viability.

EC compound synthesis

Intermediates were carried to the next step without further purification unless otherwise noted, ^{1}H NMR was used to confirm the formation of the desired product. EC compounds were characterized by ^{1}H NMR, ^{13}C NMR and HR-ESI-MS. EC compound purity was determined by UPLC-MS analysis. NMR spectrum were collected on a Bruker Avance Neo 500 MHz spectrometer or 300 MHz spectrometer, deuterated solvents were purchased from Sigma Aldrich or Tokyo Chemical Industry (TCI). NMR spectra were processed using MestReNova by Mestrelab Research S.L. Accurate mass was obtained by electrospray ionization using a Thermo Scientific™ Exactive™ Plus Orbitrap Ultimate 3000 LC-MS system, using an eluent of 50:50 Mili-Q™ water and Optima™ Acetonitrile. Ultra-performance liquid chromatography-mass spectrometry (UPLC-MS) was performed on a Waters UPLC-MS equipped

with UV/Vis and QDa detector and an Aquity UPLC BEH C18 1.7 μm (21 x 50 mm) column.

Fragment A

tert-butyl 4-(5-bromopyridin-2-yl)piperazine-1-carboxylate [A1] was purchased from Sigma-Aldrich.

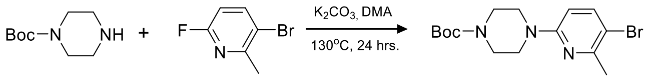

A2

tert-butyl 4-(5-bromo-6-methylpyridin-2-yl)piperazine-1-carboxylate [A2]: A round bottom flask was charged with 3-bromo-6-fluoro-2-methylpyridine (100 mg, 0.53 mmol, 1 eq.), boc-piperazine (294 mg,1.58 mmol, 3 eq.), K_2CO_3 (145 mg, 1.05 mmol, 2 eq.), and DMA (5 mL). The reaction was refluxed at 130°C for 24 hours. The reaction was then cooled and concentrate to dryness on a rotary evaporator. The crude material was dissolved in 1:1 diethyl ether/water, the organic layer was collected, and the aqueous layer was washed with diethyl ether (x2). The combined organic layer was washed with brine (x3), 5% LiCl (x3) and H_2O (x1) then dried over Na_2SO_4. The Na_2SO_4 was removed with filtration and the filtrate was concentrate to dryness on a rotary evaporator. The crude material was then dissolved in DCM and filtered through a basic alumina plug before concentrating to dryness on a rotary evaporator. Crude A2 (125 mg, 75% yield) was obtained as white solid and used as such for next steps. ^{1}H NMR (500 MHz, $CDCl_3$) δ 7.52 (d, J = 8.8 Hz, 1H), 6.34 (d, J = 8.8 Hz, 1H), 3.53 – 3.47 (m, 8H), 2.49 (s, 3H), 1.48 (s, 9H).

Fragment B.

General synthetic procedure: In a microwave vial Fragment A (1 eq.), boronic acid (4 eq.), Na_2CO_3 (4 eq.), TBAB (0.5 eq.), THF (2 mL), H_2O (2 mL) were added along with a stir bar. In some instances, SPhos (0.05 eq.) was also added as noted below. Immediately prior to capping the vessel $Pd(OAc)_2$ (0.1 eq) was added and the reaction was placed in a microwave reactor at 150°C for 5 minutes. After the reaction, the cap was removed, and DCM was added. The organic layer was extracted and washed with 1 M NaOH (x3) and then dried over Na_2SO_4. The mixture was then filtered through neutral alumina and concentrated to dryness on a rotary

evaporator to obtain crude Fragment B. Fragment B was obtained as a crude material and used as such in the next step.

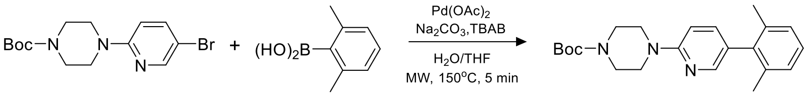

A1 B1

tert-butyl 4-(5-(2,6-dimethylphenyl)pyridin-2-yl)piperazine-1-carboxylate [B1]: Was prepared with A1 (100 mg, 0.29 mmol, 1 eq.), 2,6-dimethylphenylboronic acid (175 mg, 1.17 mmol, 4 eq.), Na_2CO_3 (124 mg, 1.17 mmol, 4 eq.), TBAB (47 mg, 0.15 mmol, 0.5 eq.), $Pd(OAc)_2$ (6.6 mg, 0.03 mmol, 0.1 eq), THF (2 mL), H_2O (2 mL). Crude B1 (107 mg).

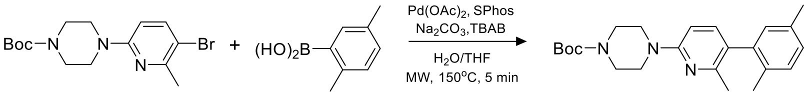

A2 B2

tert-butyl 4-(5-(2,5-dimethylphenyl)-6-methylpyridin-2-yl)piperazine-1-carboxylate [B2]: Was prepared with A2 (100 mg, 0.28 mmol, 1 eq.), 2,5-dimethylphenylboronic acd (168 mg, 1.12 mmol, 4 eq.), Na_2CO_3 (119 mg, 1.12 mmol, 4 eq.), TBAB (45 mg, 0.14 mmol, 0.5 eq.), SPhos (5.8 mg, 0.01 mmol, 0.05 eq.), $Pd(OAc)_2$ (6.3 mg, 0.03 mmol, 0.1 eq), THF (2 mL), H_2O (2 mL). Crude B2 (112 mg).

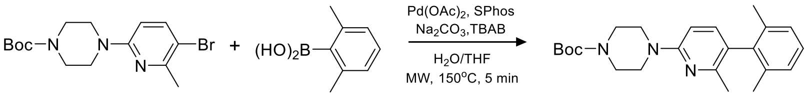

A2 B3

tert-butyl 4-(5-(2,6-dimethylphenyl)-6-methylpyridin-2-yl)piperazine-1-carboxylate [B3]: Was prepared with A2 (75 mg, 0.21 mmol, 1 eq.), 2,6-dimethylphenylboronic acid (126 mg, 0.84 mmol, 4 eq.), Na_2CO_3 (89 mg, 0.84 mmol, 4 eq.), TBAB (34 mg, 0.11 mmol, 0.5 eq.), SPhos (4.3 mg, 0.01 mmol, 0.05 eq.), $Pd(OAc)_2$ (4.7 mg, 0.02 mmol, 0.1 eq), THF (2 mL), H_2O (2 mL). Crude B3 (102 mg).

<u>Fragment C.</u>

General synthetic procedure: A round bottom flask was charged with a stir bar and Fragment B. The vessel was purged with N_2 and placed in an ice bath (0°C). 4 M HCl in dioxane was separately chilled to 0°C and then added via syringe to Fragment B and stirred for 30 min. The reaction was then concentrated to dryness on a rotary evaporator and the solid was washed with DCM and filter via vacuum filtration to obtain crude Fragment C. Fragment C was obtained as a crude material and used as such in the next step.

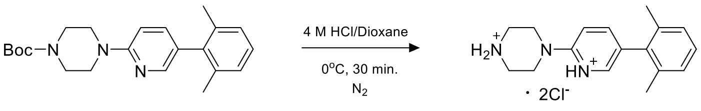

B1 **C1**

4-(5-(2,6-dimethylphenyl)pyridin-1-ium-2-yl)piperazin-1-ium [C1]: Was prepared with B1 (110 mg, 0.3 mmol, 1 eq.), 4 M HCl in dioxane (3 mL). Crude C19 (74 mg).

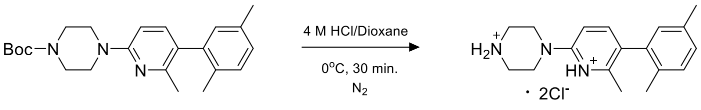

B2 **C2**

4-(5-(2,5-dimethylphenyl)-6-methylpyridin-1-ium-2-yl)piperazin-1-ium [C2]: Was prepared with B2 (112 mg, 0.29 mmol, 1 eq.), 4 M HCl in dioxane (2 mL). Crude C2 (135 mg).

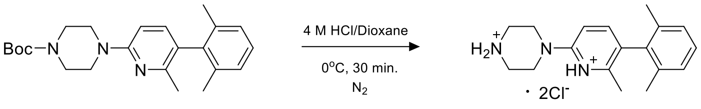

B3 **C3**

4-(5-(2,6-dimethylphenyl)-6-methylpyridin-1-ium-2-yl)piperazin-1-ium [C3]: Was prepared with B3 (102 mg, 0.27 mmol, 1 eq.), 4 M HCl in dioxane (2 mL). Crude C11 (88 mg).

<u>Fragment D.</u>

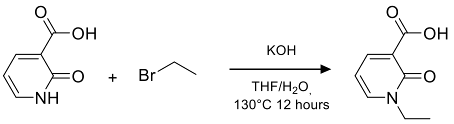

D1

1-ethyl-2-oxo-1,2-dihydropyridine-3-carboxylic acid [D1]: 1,2-dihydro-2-oxo-pyridine-3-carboxylic acid (1 g, 7.2 mmol, 1 eq.), bromoethane (2.2 mL, 30 mmol, 4 eq.) and KOH (816 mg, 14.5 mmol, 2 eq.) were stirred together in a mixture of THF (12 mL) and water (6 mL) at 130°C in a pressure flask for 12 hours. After cooling the mixture to room temperature, 80 mL water was added, and the pH was reduced to 4 using 1 M HCl. The mixture was left in the fridge (4°C) to crystallize and needle shaped crystals grew over a week which were collected and washed with water to give D1 (780 mg). ^{1}H NMR (300 MHz, MeOD) δ 8.46 (d, *J* = 7.3 Hz, 1H), 8.10 (d, *J* = 6.5 Hz, 1H), 6.68 (t, *J* = 6.9 Hz, 1H), 4.19 (q, *J* = 7.2 Hz, 2H), 1.40 (t, *J* = 7.2 Hz, 3H).

1-cyclopropyl-2-oxo-1,2-dihydropyridine-3-carboxylic acid [D2] was purchased from Enamine.

EC-Compounds.

General synthetic procedure: Combine Fragment D (1 eq.), HBTU (1 eq.), DIPEA (4 eq.), and dry DMF (1 mL/10 mg Fragment D) in an oven-dried vessel at 0°C under N_2, stir for 30 min. Fragment C (1 eq.) was dissolved in 3 mL of dry DMF at 0°C and added to the solution containing Fragment D. Reaction was stirred under N_2 overnight (0°C to rt). The reaction was then concentrated to dryness on a rotary evaporator and redissolved in DCM. 5% LiCl was added and the organic layer was washed with 5% LiCl (x2), H_2O (x1), and brine (x1), then dried over Na_2SO_4. Na_2SO_4 was removed with filtration and the filtrate was concentrated to dryness on a rotary evaporator. Compounds were purified via flash chromatography, basic alumina (Act I) using 0-1% MeOH/DCM gradient eluent.

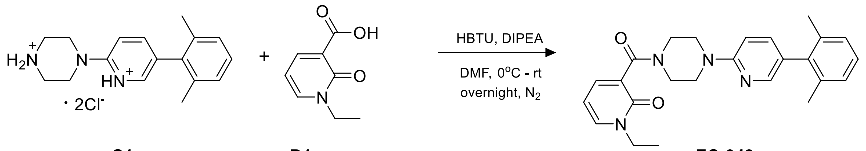

C1 **D1** **EC-049**

3-(4-(5-(2,6-dimethylphenyl)pyridin-2-yl)piperazine-1-carbonyl)-1-ethylpyridin-2(1H)-one [EC-049]: Prepared with D1 (25 mg, 0.15 mmol, 1 eq.), HBTU (57 mg, 0.15 mmol, 1 eq.), DIPEA (77 mg, 104 µL, 0.60 mmol, 4 eq.), C1 (53 mg, 0.15 mmol, 1 eq.), DMF (6 mL). EC-049 was obtained (28 mg, 45% yield, 92% pure). ^{1}H NMR (500 MHz, $CDCl_3$) δ 7.99 (d, *J* = 2.4 Hz, 1H), 7.56 (dd, *J* = 6.9, 2.1 Hz, 1H), 7.39 (dd, *J* = 6.7, 2.1 Hz, 1H), 7.31 (dd, *J* = 8.6, 2.4 Hz, 1H), 7.17 – 7.09 (m, 3H), 6.73 (d, *J* = 8.6 Hz, 1H), 6.26 (t, *J* = 6.8 Hz, 1H), 4.03 – 4.01 (m, 2H), 3.94 – 3.81 (m, 2H), 3.70 – 3.66 (m, 4H), 3.51 – 3.49 (m, 2H), 2.06 (s, 6H), 1.36 (t, *J* = 7.2 Hz, 3H). ^{13}C NMR (126 MHz, $CDCl_3$) δ 166.3, 158.9, 158.1, 147.9, 139.7, 138.8, 138.8, 138.4, 137.1, 128.5, 127.5, 127.4, 126.5, 107.0, 105.9, 46.7, 45.8, 45.6, 45.3, 41.9, 21.1, 14.7. HR-ESI-MS: m/z $[M+H]^+$ calculated for $C_{25}H_{29}N_4O_2^+$: 417.22851; found: 417.22850.

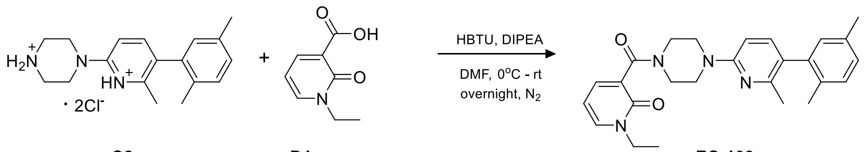

C2 **D1** **EC-103**

3-(4-(5-(2,5-dimethylphenyl)-6-methylpyridin-2-yl)piperazine-1-carbonyl)-1-ethylpyridin-2(1H)-one [EC-103]: Prepared with D1 (25 mg, 0.15 mmol, 1 eq.), HBTU (57 mg, 0.15 mmol, 1 eq.), DIPEA (77 mg, 104 µL, 0.60 mmol, 4 eq.), C2 (53 mg, 0.15 mmol, 1 eq.), DMF (6 mL). EC-103 was obtained (12 mg, 19% yield, >95%

pure). ^{1}H NMR (300 MHz, $CDCl_3$) δ 7.57 (dd, *J* = 6.9, 2.0 Hz, 1H), 7.39 (dd, *J* = 6.8, 2.1 Hz, 1H), 7.25 – 7.21 (m, 1H), 7.16 – 7.13 (m, 1H), 7.07 – 7.04 (m, 1H), 6.90 (s, 1H), 6.52 (d, *J* = 8.4 Hz, 1H), 6.26 (t, *J* = 6.8 Hz, 1H), 4.03 (q, *J* = 7.2 Hz, 2H), 3.94 – 3.90 (m, 2H), 3.68 – 3.65 (m, 4H), 3.54 – 3.50 (m, 2H), 2.32 (s, 3H), 2.16 (s, 3H), 2.03 (s, 3H), 1.38 (t, *J* = 7.2 Hz, 3H). ^{13}C NMR (126 MHz, DMSO-d_6) δ 165.6, 158.2, 157.3, 152.9, 140.0, 139.5, 138.9, 138.8, 134.7, 132.6, 130.3, 129.8, 127.9, 127.5, 125.4, 105.2, 104.3, 46.0, 45.0, 44.4, 44.3, 41.1, 22.6, 20.6, 19.2, 14.5. HR-ESI-MS: m/z [*M*+H]$^+$ calculated for $C_{26}H_{31}N_4O_2^+$: 431.24416; found: 431.24416.

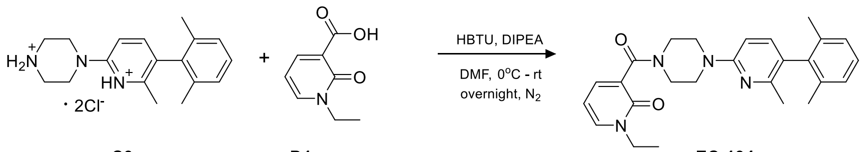

C3 D1 EC-134

3-(4-(5-(2,6-dimethylphenyl)-6-methylpyridin-2-yl)piperazine-1-carbonyl)-1-ethylpyridin-2(1H)-one [EC-134]: Prepared with D1 (25 mg, 0.15 mmol, 1 eq.), HBTU (57 mg, 0.15 mmol, 1 eq.), DIPEA (77 mg, 104 µL, 0.60 mmol, 4 eq.), C3 (53 mg, 0.15 mmol, 1 eq.), DMF (6 mL). EC-134 was obtained (32 mg, 50% yield, >95% pure). ^{1}H NMR (300 MHz, $CDCl_3$) δ 7.56 (dd, *J* = 6.9, 2.1 Hz, 1H), 7.39 (dd, *J* = 6.7, 2.1 Hz, 1H), 7.17 – 7.07 (m, 4H), 6.56 (dd, *J* = 8.5, 0.7 Hz, 1H), 6.26 (t, *J* = 6.8 Hz, 1H), 4.02 (q, *J* = 7.2 Hz, 2H), 3.94 – 3.91 (m, 2H), 3.69 – 3.63 (m, 4H), 3.52 – 3.48 (m, 2H), 2.07 (s, 3H), 1.97 (s, 6H), 1.37 (t, *J* = 7.2 Hz, 3H). ^{13}C NMR (126 MHz, DMSO-d_6) δ 165.5, 158.1, 157.3, 152.7, 139.9, 139.1, 138.8, 138.6, 135.9 (x2), 127.5, 127.3 (x2), 127.1, 123.9, 105.1, 104.7, 45.9, 44.9, 44.3, 44.2, 41.1, 22.1, 20.2 (x2), 14.4. Symmetrical carbons determined by HSQC and HMBC. HR-ESI-MS: m/z [*M*+H]$^+$ calculated for $C_{26}H_{31}N_4O_2^+$: 431.24416; found: 431.24414.

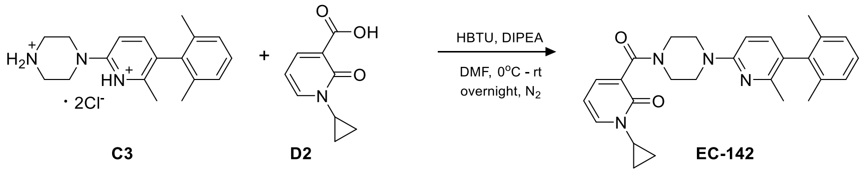

C3 D2 EC-142

1-cyclopropyl-3-(4-(5-(2,6-dimethylphenyl)-6-methylpyridin-2-yl)piperazine-1-carbonyl)pyridin-2(1H)-one [EC-142]: Prepared with D2 (25 mg, 0.14 mmol, 1 eq.), HBTU (53 mg, 0.14 mmol, 1 eq.), DIPEA (72 mg, 98 µL, 0.56 mmol, 4 eq.), C3 (49 mg, 0.14 mmol, 1 eq.), DMF (6 mL). EC-142 was obtained (52 mg, 84% yield, >95% pure). ^{1}H NMR (500 MHz, $CDCl_3$) δ 7.53 (dd, *J* = 6.8, 2.1 Hz, 1H), 7.40 (dd, *J* = 6.9, 2.1 Hz, 1H), 7.15 – 7.12 (m, 2H), 7.09 – 7.07 (m, 2H), 6.55 (d, *J* = 8.4 Hz, 1H), 6.21

(t, J = 6.8 Hz, 1H), 3.92 – 3.90 (m, 2H), 3.68 – 3.65 (m, 4H), 3.51 – 3.49 (m, 2H), 2.06 (s, 3H), 1.96 (s, 6H), 1.17 – 1.13 (m, 2H), 0.86 – 0.84 (m, 2H). ^{13}C NMR (126 MHz, $CDCl_3$) δ 166.1, 160.5, 158.1, 154.2, 139.4, 139.3, 138.8, 138.7, 136.7, 128.4, 127.4, 127.2, 125.4, 105.3, 104.6, 46.8, 46.0, 45.4, 41.9, 32.7, 22.4, 20.6, 7.0. HR-ESI-MS: m/z $[M+H]^+$ calculated for $C_{27}H_{31}N_4O_2^+$: 443.24416; found: 443.24415.

CBX7-chromodomain protein expression

CBX7-chromodomain was expressed with an N-terminal His6-tag and a TEV protease cleavage site using Addgene plasmid 25241 (from the lab of C. Arrowsmith, Structural Genomics Consortium. Toronto, Canada). BL21 Codon Plus RIL Eschericia coli cells (Stratagene) were transformed with this plasmid and selected against Kanamycin. The bacterium was cultured at 37°C in 2XYT media till O.D. ≥ 1.7. The temperature was dropped to 15°C for one hour before inducing them with 1 mM IPTG (Life Technologies). The cells were incubated overnight before pelleting and resuspending in “binding buffer” (20 mM Tris-HCl, 20 mM Imidazole, 150 mM NaCl, pH 7.4) and spiked with Protease Inhibitor (Pierce Protease Inhibitor Mini Tablets ETDA Free, Thermo Scientific Catalog number A32955, 1 tablet per 10 mL buffer used). The pellets can be flash-frozen at this point and stored until ready for purification.

CBX7-chromodomain protein purification

The night before purification, if flash-frozen, the pellets were thawed on ice at 4°C. The pellets were lysed by first incubating them with CHAPS solution (50 µL of 0.1 mg/mL of CHAPS solution in dH_2O for every mL of resuspended pellets) for 30 minutes at room temperature. After that, the pellets were sonicated using an ultrasonicator in short bursts (four 10-second bursts with ~30-45-second wait in between) on ice. Cell debris was removed using ultracentrifugation, the supernatant was purified immediately. Purification of CBX7 was done in three sequential steps. First, an affinity chromatography step using a Ni-NTA chelating column (HisTrap HP, GE) was done. The column was hooked to an AKTA Plus Liquid Chromatography System (GE) and primed with “binding buffer” before loading the supernatant from the step above. The column was washed with the “binding buffer” till the UV response went below 20 mAU. CBX7 was then eluted by slowly replacing “binding buffer” with “elution buffer” (20 mM Tris-HCl, 150 mM NaCl, 500 mM Imidazole, pH

7.4) in a 60-minute long gradient. The peak with the biggest UV response was collected and immediately moved to a 4°C fridge. Next, the his-tag was cleaved using in-house-grown TEV proteases. This was done by first concentrating and simultaneously buffer-exchanging fractions from the previous step into the "dialysis buffer" (50 mM Tris-HCl, 150 mM NaCl, 0.6 mM EDTA, 1 mM DTT, pH 7.4) using Amicon spin concentrator tube (Millipore Sigma) at 4°C till the volume was ~ 5 mL. The concentration of protein was estimated using its A280 (Extinction coefficient - 22460). TEV protease was added in a ratio of 1:50 with respect to the protein concentration and the solution was incubated overnight at 4°C. The final step was an FPLC that simultaneously purified and buffer exchanged CBX7 into minimal FP buffer (20 mM Tris-HCl, 250 mM NaCl, 0.01% Tween-20, pH 8.0). This was done using a Hiload 16/600 Superdex 75pg size exclusion column (GE) setup on an AKTA Plus Liquid Chromatography System (GE). The running buffer (same as minimal FP buffer) was thoroughly degassed before use. The fractions were collected based on their UV response and concentrated using Amicon spin concentrator tubes (Millipore Sigma) at 4°C till the desired concentration of protein was reached (estimated using A280, Extinction coefficient - 20970). The entire expression and purification were followed using 15 % SDS PAGE (stained using Coomassie Blue). His-tag removal was confirmed by the decrease in molecular weight of the protein band.

Competitive fluorescence polarization (FP)

Was performed as previously reported (2). In summary, a previously reported FITC-tagged peptide (P1, compound 2 in reference 26.) was used as the competitive binding probe. Compounds were dissolved in 100% DMSO and then diluted into full FP buffer. Full FP buffer was made from the addition of DTT, PMSF, and benzamidine (all 1 mM final concentration) into minimal FP buffer (20 mM Tris-HCl, 250 mM NaCl, 0.01% Tween-20). Final concentration of DMSO was kept at 5%. The assay was carried out in NUNC black 96-well plates. Wells contained CBX7 (0.2 μM), P1 (100 nM) and inhibitor (0 – 1 mM) in full FP buffer. All wells in the plate were made to a final volume of 100 μL. The plate was centrifuged 1200 rpm x 1 min, then incubated for 15 minutes in the dark. The plate was read on a BioTek Cytation 5 cell imaging and multi-mode reader plate reader. A polarized filter cube was used with filters for excitation at 485 nm and emission at 520 nm (with a cut-off of 510 nm). Competitive FP was analyzed as previously reported 26. In summary, the buffer

blank was subtracted, and the data was fitted using a function that extrapolates the IC_{50} value in GraphPad Prism 9. Experiments were conducted in technical triplicates. Error reported as asymmetrical 95% confidence intervals.

Treatment with CBX7 or EHMT1/2 inhibitors in leukemic cell lines

For inhibitor treatment, 250 000 leukemic cells/mL were cultured in their appropriate culture medium supplemented with increasing concentrations of the CBX7-inhibitors MS452 (Sigma, SML1405), EC-134 (synthesized in this study), or BDA-41 (gift from B. Zhang (3)) or with increasing concentrations of the EHMT1/2 inhibitor UNC0642 (Sigma, SML1037). All compounds were dissolved in DMSO and added to the cells as 0.1% DMSO solution. After four days of treatment, cells were counted manually using a hemocytometer to assess viability. IC_{50} values were obtained by using the GraphPad Prism's built-in normalized model (with settings; [Inhibitor] vs normalized response – variable slope).

Flow cytometric analysis of histone modification

Four days post-treatment, 200 000 OCI-AML3 cells were harvested and resuspended in PBS. To distinguish between live and dead cells, the fixable Zombie Violet dye (1:1000) (Biolegend, 423113) was added to the cell suspension and incubated for 20 minutes at RT. After washing (400g, 5 min., 4°C) with PBS, the cells were fixed in ice-cold 70% EtOH for 1 hour at 4°C. After fixation, cells were washed and resuspended in 0.05% Tween-PBS to keep the cell membrane permeable. To prevent unspecific binding of the antibodies to Fc receptors, 1% Fc block (BD, #564220) was added to the cells and incubated for 10 minutes at RT. Subsequently, cells were stained with AlexaFluor488-labeled mouse-anti-H3K9me2 (1:1000) (Abcam, ab203850, RRID:AB_2890248) and with rabbit-anti-Ubiquityl-Histone H2A (Lys119) (1:1000) (Cell signaling, #8240, RRID:AB_10891618) conjugated to AlexaFluor647 using the Zenon labeling kit (Invitrogen, Z25308) (1 uL antibody was conjugated using 3.2 µL Zenon labeling reagent and 3.2 µL Zenon IgG blocking solution). After 1 hour incubation at RT, cells were first washed with 0.05% Tween-PBS and subsequently washed with PBS before measurement on Canto II (BD).

Retroviral transduction of the CBX-GFP fusion constructs into OCI-AML3 cells

200 000 Plat-A cells/well were seeded in a 6-well plate in their standard DMEM culture medium. After one day of culture, the plat-A cells were transfected with the retroviral CBX-GFP constructs (gift from Dr. V. van den Boom (4, 5)) by incubating 2 ug CBX-GFP plasmid, 7 µL FuGENE (Promega, E2311) and 150 µL1 opti-MEM medium (Gibco, 31985062) for 10 minutes at RT followed by adding the mixture to the cells. After one day of culture, medium was replaced by OCI-AML3 standard RPMI medium. After another day of culture, RPMI medium with the viral supernatant was harvested and filtered using a Sterile Syringe filter unit, PVDF, 0.45 um, before spinfecting (400g for 45 min, RT) together with 200 000 OCI-AML3 cells on a retronectin (Takara Bio, T100) pre-coated 6 wells plate. After three days of culture, cells were harvested, propidium iodide (Invitrogen, P1304MP) was added and GFP+PI- OCI-AML3 cells were sorted using the 100 µm nozzle on the Sony SH800S cell sorter.

Chromatin immunoprecipitation (ChIP)-qPCR

For ChIP experiments, $4 * 10^6$ stable CBX-GFP expressing OCI-AML3 cells were cultured in their standard RPMI medium and exposed to DMSO only, or to 100 µM MS452, 100 µM EC-134 or 35 µM BDA-41 dissolved in DMSO. After 24 hours of culture, cells were harvested and washed with 0.2% BSA-PBS (450g, 5 minutes) and fixated with 1% formaldehyde while gently rotated at 4°C for 10 minutes, followed by terminating the fixation with the addition of 125 µM glycine for an additional 5 minutes still rotating at 4°C. Cells were washed twice with PBS (450g, 5 minutes) before they were resuspended in 500 µL SDS lysis buffer (100 mM NaCl, 50 mM Tris-HCL pH 8.1, 5 mM EDTA pH 8.0, 0.02% NaN_3, 0.5% SDS in ultra-pure H_2O) supplemented with proteinase inhibitors (Roche, #11873580001) (1 tablet per 50 mL buffer). After ≥ 1 hour storage at -80°C, lysates were centrifugated for 5 minutes at 1200g whereafter they were resuspended in 300 µL ice-cold IP buffer (100 mM NaCl, 33.3 mM Tris-HCL pH 8.1, 33.3 mM Tris-HCL pH 8.6, 5 mM EDTA pH 8.0, 0.02% NaN_3, 0.33% SDS, 1.67% Triton X-100 in ultra-pure H_2O) supplemented with proteinase inhibitor. DNA was fragmented to an average length of 250-750 bps by sonicating the cell lysates with a Bioruptor Plus (Diagenode, B01020001) with the settings 30s ON, 30s OFF, high for 4 cycles. After ≥ 1 hour of storage at -80C, the sonicated samples were centrifugated for 30 minutes at 17000g, 4°C. To prevent

unspecific binding of the Protein G immunobeads (Invitrogen, 10004D), samples were pre-cleared by incubating the samples with 20 μL immunobeads while gently rotating at 4°C for ≥ 1 hour. A magnetic stand was used to collect the cleared samples whereafter the samples were divided into three conditions namely, 13.5 μL for input, 135 μL for GFP(-CBX), and 135 μL for IgG control. The input sample was stored at 4°C before further use. For antibody incubation, the remaining two conditions were incubated with 2 ul rabbit-anti-GFP (Abcam, ab290, RRID:AB_303395) or 2 μL 5 mg/mL Rabbit IgG control (Sigma, I5006) overnight at 4°C. To get rid of aggregates formed overnight, samples were centrifugated for 30 minutes at 17000g, 4°C, and the supernatant was collected in a new tube. Samples were blocked with 75ng/μL ssDNA (Sigma, D1626) and 0.1% BSA for ≥ 15 minutes at 4°C while gently rotating before samples were incubated with 50 μL immunobeads for ≥ 2 hours at 4°C while still rotating. Using a magnetic stand, the bead-sample complexes were washed two times with cold low salt buffer (150 mM NaCl, 20 mM Tris-HCL pH 8.1, 5 mM EDTA pH 8.0, 5.2% sucrose, 0.02% NaN_3, 1% Triton X-100, 0.2% SDS in ultra-pure H_2O), four times with cold LiCl-detergent buffer (0.5% deoxycholic acid, 1 mM EDTA pH 8.0, 250 mM LiCl, 0.5% NP-40, 10 mM Tris-HCL pH 8.0, 0.02% NaN_3 SDS in ultra-pure H_2O), two times with cold low salt buffer, and finally one time with cold TE buffer. Washed bead-sample complexes were reversed cross-linked and eluted in elution buffer (90 mM $NaHCO_3$, 1% SDS in ultra-pure H2O) by heating at 65°C for 1-2 hours, 1000rpm. Beads were removed using a magnetic stand followed by adding 0.2 ug/mL Proteinase K (Qiagen, #19131) to the eluted samples, as well as to the input samples, and incubated for 2 hours at 65°C, 1000rpm. DNA was purified using the MinElute PCR Purification kit (Qiagen, #28004), and purified DNA was used for qPCR.

Quantitative PCR (qPCR)

Total RNA was extracted from OCI-AML3 cells four days post-treatment or from freshly thawed primary cells in RLT buffer according to manufacturing’s instructions (Qiagen, #74034 or #74134). Extracted RNA was used to synthesize cDNA according to manufacturing’s instructions (Invitrogen, #K1621).

ChIP-DNA or cDNA was applied for qPCR using LightCycler SYBR Green MasterMix (Roche, #4707516001) in a LightCycler 480 system (Roche). Primers are listed in the Table S3.

Flow cytometric cell cycle analysis

Four days post-treatment, 200 000 OCI-AML3 cells were harvested, washed with cold PBS (450g, 5 minutes), and resuspended in cold 70% EtOH for 1 hour at 4°C to fixate the cells. Cells were washed with cold PBS (450g, 5 minutes) and resuspended in 0.05% Tween-PBS to keep the cell membrane permeable. Cells were blocked by incubating the cells for 10 minutes at RT with 1% Fc block after which cells were incubated with AlexaFluor488-labeled Ki-67 (1:100) (BD, #558616, RRID:AB_647087) for 1 hour at RT. After staining, cells were washed with 0.05% Tween-PBS before resuspended in 2 ug/ul DAPI (BD, #564907). Cells were measured on Canto II.

Cell tracer based proliferation assays

At day zero, cells were washed with PBS (450g, 5 minutes) before 1 * 106 OCI-AML3 cells/mL or 200 000 Nalm-6 cells/mL were stained with, 5 µM or 1 µM respectively, Cell Tracer Violet proliferation dye (Invitrogen, C34557) for 20 minutes at 37°C. To stop the incubation, serum was added and cells were washed (450g, 5 minutes) before 250 000 cells/mL were resuspended in their appropriate culture medium supplemented with inhibitors as described above. Propidium iodide was added before cells from day zero and day four were measured on Canto II.

Flow cytometric differentiation analysis

Four days post-treatment, 50 000-200 000 OCI-AML3, EOL-1, or Nalm-6 cells (with equal cell numbers within the same experiment) were harvested and washed with 0.2% BSA-PBS. To prevent unspecific binding of the antibodies to Fc receptors, 1% Fc block was added to the cells and incubated for 10 minutes at RT. After blocking, cells were stained with either BV421-labeled CD11b (1:100) (BD, #562632, RRID:AB_2737689), BV421-labeled CD20 (1:100) (BD, #562873, RRID:AB_2737857), APC-labeled CD10 (1:100) (Biolegend, #312209, RRID:AB_314920), AlexaFluor700-labeled CD14 (1:100) (BD, #557923, RRID:AB_396944), or a combination of antibodies for 20 minutes at 4°C. Cells were washed with 0.2 % BSA-PBS (450g, 5 minutes) before propidium iodide was added. Cells were measured on Canto II.

May-Grünwald Giemsa staining

Four days post-treatment, OCI-AML3 cells were cytospun on microscope slides and air-dried. Dried slides, were incubated in filtered May-Grünwald staining solution for 5 minutes whereafter slides were washed in PBS for 90 seconds. Subsequently, slides were stained in filtered 20x diluted Giemsa staining solution for 15 minutes whereafter slides were rinsed in deionized H2O. Slides were air-dried before pictures were taken using an Olympus BX43 microscope with Olympus XC30 camera.

Flow cytometric apoptosis analysis

Four days post-treatment, 30 000-50 000 OCI-AML3 and EOL-1 cells were harvested, washed with 0.2% BSA-PBS (450g, 5 minutes), and resuspended in 1x Annexin binding buffer (BD, #556454) supplemented with BV421-labeled AnnexinV (BD, #563973, RRID:AB_2869538) (1:100) and 7-AAD (Biolegend, #420404) (1:100) and incubated for 15 minutes at RT. Cells were further diluted with 1x Annexin binding buffer before measured on Canto II.

Short-term CBX7-inhibitor treatment in primary samples

For short-term CBX7-inhibitor treatment, 250 000-500 000 primary AML, ALL or CD34+ CB cells/mL were cultured in their appropriate short-term culture medium supplemented with DMSO alone, 50 µM MS452, 32 µM EC-134 or 8 µM BDA-41. After one week of treatment, depending on the cell confluence, 50-200 µL cell suspension was harvested, not washed, and applied to absolute counting tubes (BD, #663028). Subsequently, cells were blocked with 2% Fc block for 10 minutes. AML cells, and their experimental healthy CB control cells, were stained with BV421-labeled CD11b (1:250), FITC-labeled (1:50), or BB515-labeled CD38 (1:250) (BD, #555459, RRID:AB_395852 or #564498, RRID:AB_2744374), APC-labeled CD45 (1:250) (BD, #555485, RRID:AB_398600), and PeCy7-labeled CD34 (1:250) (BD, #348811, RID:AB_2868855) or CD117 (1:250) (Biolegend, #313212, RRID:AB_893222) for 20 minutes at 4°C. ALL cells were stained with BV421-labeled CD20 (1:250), FITC-labeled CD38 (1:50), PE-labeled CD19 (1:250) (BD, #561741, RRID:AB_10893795), APC-labeled CD10 (1:250), and PeCy7-labeled CD34 (1:250) for 20 minutes at 4°C. After staining, 7-AAD was added before cells were measured on Canto II. For AML experiments, the absolute cell count of CD45dim cells was

determined to assess the viability of blast and progenitor cells. For ALL experiments, the absolute cell count of CD19+ was determined to assess the viability of ALL cells.

Long-term CBX7-inhibitor treatment in primary samples

Long-term CBX7-inhibitor treatments were performed in the same way as short-term treatments, but cells were cultured in their appropriate long-term culture medium. Each week, after determining absolute cell count, cells were diluted, medium and compounds were refreshed and cells were replated. Absolute cell count was used to calculate the cumulative cell count over time.

Xenotransplantation of *ex vivo* CBX7-inhibitor treated AML cells

Primary AML cells were thawed and cultured in short-term culture medium supplemented with 50 µM MS452 or 8 µM BDA-41. After 24 hours, cells were harvested, washed twice with 2mM EDTA-, 0.2% BSA-PBS (800g for 5 minutes, followed by 200g for 10 minutes), and resuspended in 300 µL 2mM EDTA-, 0.2% BSA-PBS. To prevent graft-versus-host reactions, the CD3+ fraction was depleted by incubating the cells with 100 µL magnetic CD3 microbeads (Miltenyi Biotec, 130-050-101 and FCR blocking reagent for 30 minutes at 4°C. Cells were washed with 2mM EDTA-, 0.2% BSA-PBS (450g for 5 minutes) before the cells were applied to an LS column and the flowthrough CD3- cells were collected. CD3- cells were washed, live cells were counted manually with a hemocytometer, and cells were resuspended in RPMI medium. Equal CD3- cell numbers (500 000 AML4 cells/mouse, $1.7 * 10^6$ AML5 cells/mouse, 200 000 AML8 cells/mouse, and 37 000 AML9 cells/mouse) were intra-venously injected in irradiated NSG mice.

Peripheral blood analysis of PDX mice

From 4-5 weeks post-transplantation, every two or three weeks, peripheral blood (PB) was obtained from NSG mice under general anesthesia via retro-orbital bleeding. Absolute white blood cell (WBC) count was determined using Smart V5 (Menarini diagnostics). The remaining blood was lysed in ery-lysis buffer (156 mM NH4Cl, 127 µM EDTA, 17 mM NaCl), washed with 0.2% BSA-PBS, and blocked with 1% Fc block for 10 minutes at RT. The percentage human CD45+ AML cells out of all WBCs was determined by staining the cells with BV605-labeled mCD45 (1:100) (Biolegend, #110737, RRID:AB_11204076) and AlexaFluor700-labeled hCD45

(1:100) (Biolegend, #304024, RRID:AB_493760) for 20 minutes at 4°C. Cells were washed with 0.2 % BSA-PBS (450g, 5 minutes) before propidium iodide was added. Cells were measured on Canto II.

Bone marrow and spleen analysis of PDX mice

Eight to 14 weeks after transplantation, mice were euthanized and femurs, tibia's, and spleen were harvested. For BM analysis, one femur and two tibia's were crushed, lysed with ery-lysis, washed (450g, 5 minutes), and resuspended in 0.2% BSA-PBS. For spleen analysis, the back of a syringe plunger was used to pass spleen cells as single cells through a cell strainer. Subsequently, cells were lysed with ery-lysis, washed (450g, 5 minutes), and resuspended in 0.2% BSA-PBS. Smart V5 was used to determine the absolute WBC count in the spleen and the absolute count of hematopoietic progenitors in BM. To determine the percentage of human CD45+ AML cells out of all progenitors, $1 * 10^6$ BM cells were blocked with 1% Fc block for 10 minutes at RT and stained with BV605-labeled mCD45 (1:100), APC-labeled hCD45 (1:100), and A700-labeled mTer119 (Biolegend, #116220, RRID:AB_528963) for 20 minutes at 4°C. The percentage of hCD45+ AML cells in the spleen was determined in the same way as in PB. Cells were washed with 0.2 % BSA-PBS (450g, 5 minutes) before propidium iodide was added. Cells were measured on Canto II.

Quantification and statistical analysis

Data was plotted into graphs by using GraphPad Prism. All flow data was analyzed by using the Flowjo software (BD). Schematic figures were created by using Biorender.

Statistical details for each experiment are indicated in the legend of each figure, where appropriate.

Data availability

All data needed to evaluate the conclusions in this paper are present in the paper and/or supplementary materials. Any information required to reanalyze the data reported in this paper is available from the corresponding author upon request.

SUPPLEMENTARY FIGURE LEGENDS

Fig. S1. CBX7 DNA sequence of OCI-AML3-CBX7$^{+/-}$ cells code for a truncated CBX7 protein. (A) Alignment of the reference CBX7 DNA sequence and the CBX7 sequence in CBX7^{WT} or CBX7$^{+/-}$ OCI-AML3 cells. **(B)** Alignment of the amino acid sequence in CBX7^{WT} or CBX7$^{+/-}$ OCI-AML3 cells.

Fig. S2. EC compound synthesis and competitive fluorescence polarization data. (A) General synthetic scheme for the synthesis of EC compounds. **(B)** NMR characterization data of EC-134; top panel 1H NMR spectrum of EC-134 (300 MHz, 300 K, in CDCl3), bottom panel 13C NMR spectrum of EC-134 (126 MHz, 298 K DMSO-d6). **(C)** Competitive fluorescence polarization data of EC compounds with CBX7. EC-049; logIC50= –4.249 M, 95% CI –4.292 M to –4.205 M, R2= 0.997. EC-103; logIC50= –4.274 M, 95% CI –4.304 M to –4.244 M, R2= 0.998. EC-134; logIC50= –4.475 M, 95% CI –4.553 M to –4.396 M, R2= 0.991. EC-142; logIC50= –4.454 M, 95% CI –4.522 M to –4.385 M, R2= 0.993. Error bars are reported as asymmetrical 95% confidence intervals.

Fig. S3. CBX7-inhibitors reduce the viability of acute lymphoblastic leukemia cell lines. Percentage viable cells normalized to their experimental untreated DMSO-control of three lymphoblastic leukemic cell lines after treatment with increasing doses of the CBX7-inhibitors, MS452, and BDA-41 for four days. Data points are plotted as the mean of ≥3 replicates with SEM as error bar.

Fig. S4. CBX7-inhibitors change the epigenetic landscape of OCI-AML3 cells to an active state in a CBX7-specific context. (A) Gating strategy of histone flow experiments is shown in Figure 3A-B. MFI of H2Aub-AF647 or H3K9me2-AF488 is obtained from the Doublet-/Doublet--/Live OCI-AML3 cell population. **(B-C)** Quantification of the MFI of **(B)** H2Aub-AF647 or **(C)** H3K9me2-AF488 normalized to the DMSO control in OCI-AML3 cells treated for four days with different concentrations of the EHMT1/2 inhibitor UNC0642. Bars represent the mean of ≥3 replicates with SEM as error bar. TTEST was used to calculate the p-value between DMSO and treatment, *p<0.05, ns = not significant p>0.05. **(D)** Percentage viable OCI-AML3 cells, normalized to the DMSO control, after four days of treatment with

increasing doses of the EHMT1/2 inhibitor UNC0642. Data points are plotted as the mean of 25 replicates with SEM as error bar. **(E)** Fold change of downregulated genes in CD34+ CB cells overexpressing CBX7 compared to CD34+ CB cells transduced with the empty vector. Data is obtained from our previously published study on CBX7-overexpressing CD34+ CB cells (Jung et al., 2019). Red bars indicate cell cycle arrest-associated genes and blue bars indicate differentiation-associated genes. **(F)** ChIP-seq tracks of CBX7 in CBX7-overexpressing CD34+ CB cells demonstrating the cell cycle associated (red) and differentiation (blue) associated target genes of CBX7. Data is obtained from our previously published study on CBX7-overexpressing CD34+ CB cells (Jung et al., 2019). **(G)** CBX7, CBX2, CBX4, CBX6, and CBX8 binding to the chromatin at the p21 locus after 24 hours of treatment in OCI-AML3 overexpressing the fusion proteins CBX7-GFP, CBX2-GFP, CBX4-GFP, CBX6-GFP or CBX8-GFP. ChIP-qPCR experiments were performed to calculate %input which was used to normalize the CBX binding in the treated samples to their experimental DMSO control – see table S1 for raw data. Bars represent 1 experiment or the mean of ≥2 replicates with SEM as error bar. TTEST was used to calculate the p-value between DMSO and treatment, $^*p<0.05$.

Fig. S5. CBX7-inhibitors block proliferation, induce terminal differentiation and promote apoptosis of AML and ALL leukemic cell lines. Phenotype of the myeloid cell lines OCI-AML3 and EOL-1 and the lymphoid cell lines Nalm-6 and REH after treatment with CBX7-inhibitors for four days. **(A-B)** Differentiation potential of the myeloid cell line EOL-1. **(A)** Representative histograms of the mean fluorescent intensity (MFI) of CD11b-BV421 obtained with flow cytometry in untreated DMSO control (black), and MS452 (red) treated cells. **(B)** Normalized expression of CD11b calculated by normalizing the MFI of the MS452 treated samples to the MFI of their experimental DMSO control. Data points are plotted as the mean of ≥2 replicates with SEM as error bar. **(C)** Proliferation rate of the lymphoid cell line Nalm-6. Histograms of the mean fluorescent intensity (MFI) of the Cell tracer Violet signal in cells at the start of treatment (black), and after four days without treatment (grey), or MS452 (red) treatment from two independent experiments. **(D-G)** Differentiation potential of the lymphoid cell lines **(D-E)** Nalm-6 and **(F-G)** REH. **(D and F)** Representative histograms of the mean fluorescent intensity (MFI) of CD20-BV421 and CD10-APC obtained with flow cytometry in untreated DMSO control (black),

MS452 (red), and BDA-41 (blue) treated cells. **(E and G)** Normalized expression of CD20 (solid line, round data points) and CD10 (dotted line, triangle data points) calculated by normalizing the MFI of the treated samples to the MFI of their experimental DMSO control. Lines go through the means of the individual data points of ≥2 replicates. **(H-J)** Cell death in myeloid cell lines. **(H)** Quantification of the percentage of fragmented OCI-AML3 cells, related to the representative flow plots in Figure 4A. Bars represent the mean of ≥2 replicates with SEM as error bar. TTEST was used to calculate the p-value between DMSO and treatment, *p<0.05. **(I-J)** Apoptosis induction in EOL-1 cells. **(I)** Representative flow plots of 7AAD and AnnexinV-BV421 signal indicating the gates for live, apoptotic, or dead cell populations. **(J)** Quantification of the percentage of cells in each gate. Bars represent the mean of ≥3 replicates with SEM as error bar. TTEST was used to calculate the p-value between treatment and their experimental DMSO control, *p<0.05, where green stars indicate differences in live cells, orange stars apoptotic cells, and red stars dead cells.

Fig. S6. The effect of CBX7-inhibitors on primary AML, ALL, and CD34+ CB cells. (A) CBX7 expression levels. qPCR quantification of CBX7 transcript levels in primary AML samples and in normal CD34+ CB samples. Flat lines indicate the mean. TTEST was used to calculate the p-value between AML and normal CB, * p<0.05. **(B-F)** The effect of CBX7-inhibitors on the viability and differentiation potential of primitive AML and CD34+ CB cells cultured in Stemspan enriched with SCF, TPO, and FLT3L. **(B)** Flow plots of FSC, SSC, CD45-APC, CD34/117-PeCy7, CD38-FITC signal, and histograms of the mean fluorescent intensity (MFI) of CD11b-BV421 after treatment with MS452 (red), EC-134 (purple), BDA-41 (blue) or no treatment (black) in each patient sample shown in Figure 5. To gate the most primitive cells in cases of NPM1 mutated AMLs, anti-CD117 was used instead of anti-CD34 antibodies because NPM1+ AMLs do not express CD34. **(C)** Percentage primitive CD34/117+38- and CD34/117+38- and more differentiated CD34/117-38+ and CD34/117-38- AML cells after one-week treatment with MS452, EC-134 and BDA-41. **(D-F)** Normalized expression of **(D)** CD45, **(E)** CD11b, and **(F)** CD34/CD117 calculated by normalizing the MFI of the treated samples to the MFI of their experimental DMSO control. Lines represent the median of ≥4 different AML samples. TTEST was used to calculate the p-value between the treatment and their

experimental DMSO control, *p<0.05. **(G-H)** The effect of CBX7-inhibitors on the proliferation of primary AML and CD34+ CB cells cultured in aMEM medium enriched with 25% serum, TPO, IL-3, and G-CSF. [G] Growth curves of the primary AML or CD34+ CB cells, shown by the cumulative CD45dim cell count fold change, after treatment with MS452 (red line), EC-134 (purple line), or no treatment (black line) for max. 4 weeks. **(H)** Percentage of viable immature (CD45dim) AML (red box plot) or CD34+ CB (black box plot) cells normalized to their experimental DMSO control after max. 4 weeks of *in vitro* treatment with MS452. Boxplots represent the median of ≥5 different AML or CB samples. TTEST was used to calculate the p-value between the AML and CB samples, *p<0.05. **(I-J)** The effect of CBX7-inhibitors on the cell growth of primary ALL cells cultured in StemSpan medium enriched with SCF, TPO, FLT3L, and IL-7. **(I)** Percentage of viable CD19+ ALL cells normalized to their DMSO control after one week *in vitro* treatment with MS452 (red box plot) or BDA-41 (blue box plot). Boxplots represent the median of 11 different ALL samples. **(J)** Growth curves of the ALL cells, shown by the cumulative CD19+ ALL cell count fold change, after treatment with MS452 (red line), BDA-41 (blue line), or no treatment (black line) for 3 weeks.

Supplement Figure 1

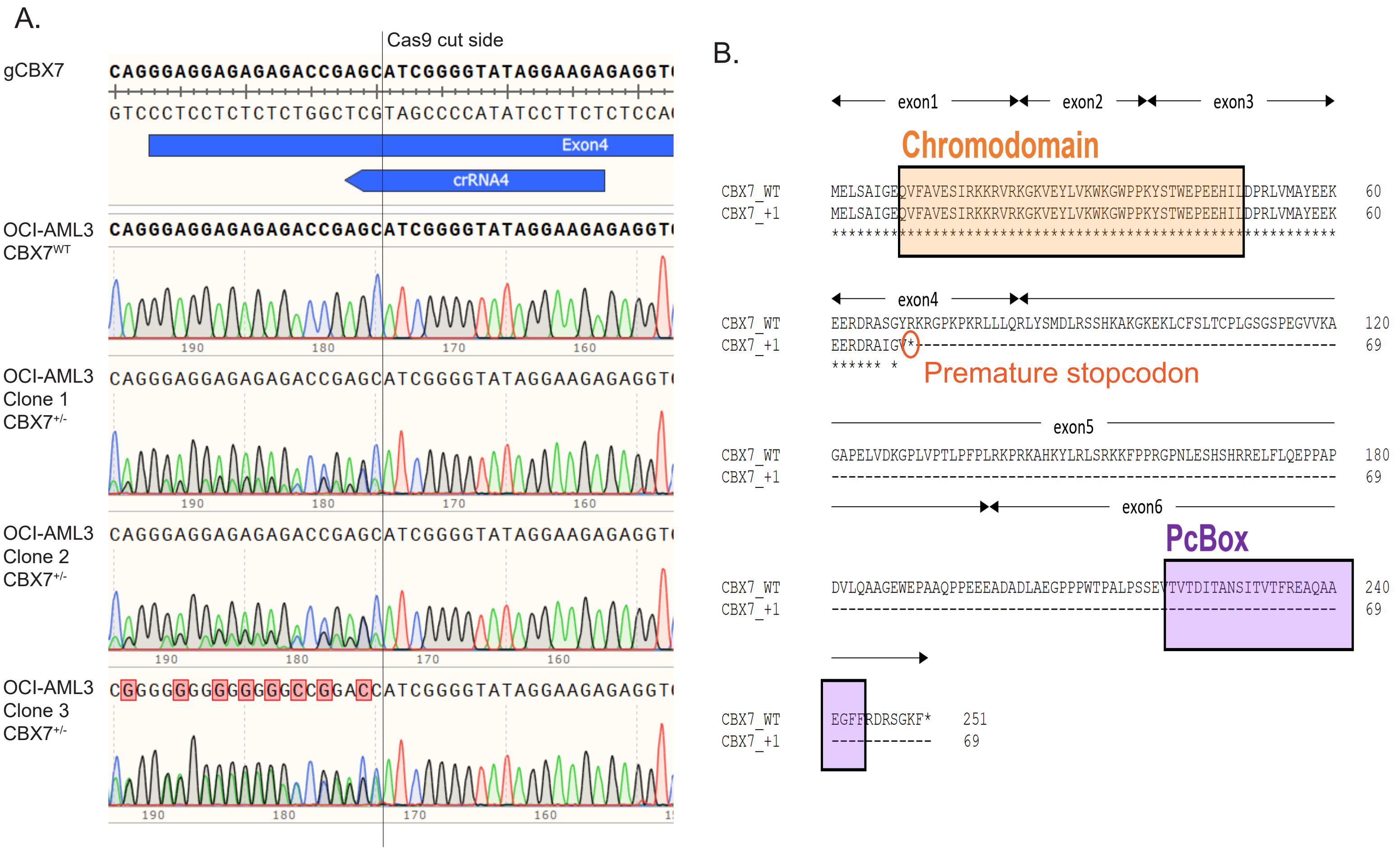

Supplement Figure 2

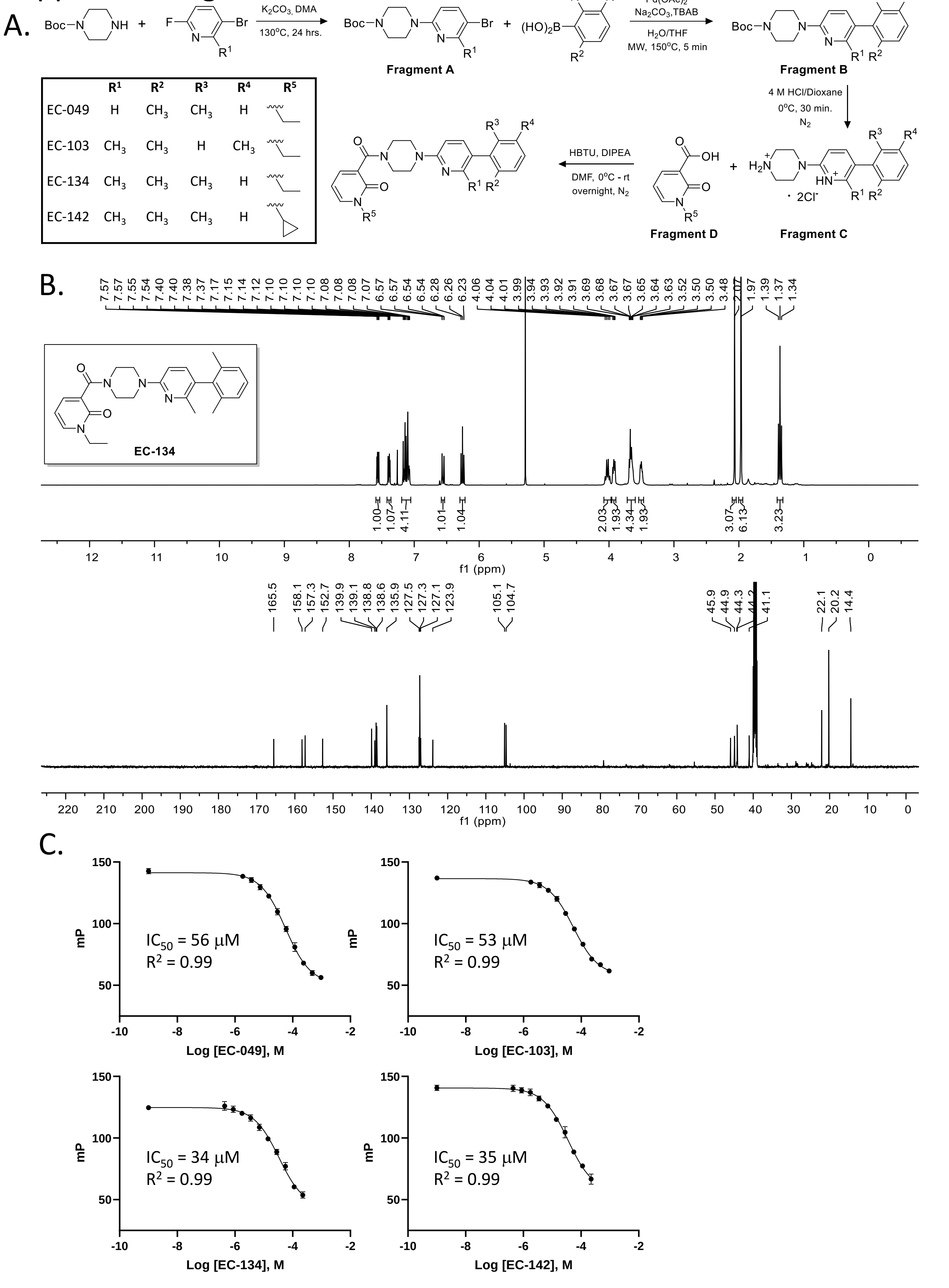

Supplement Figure 3

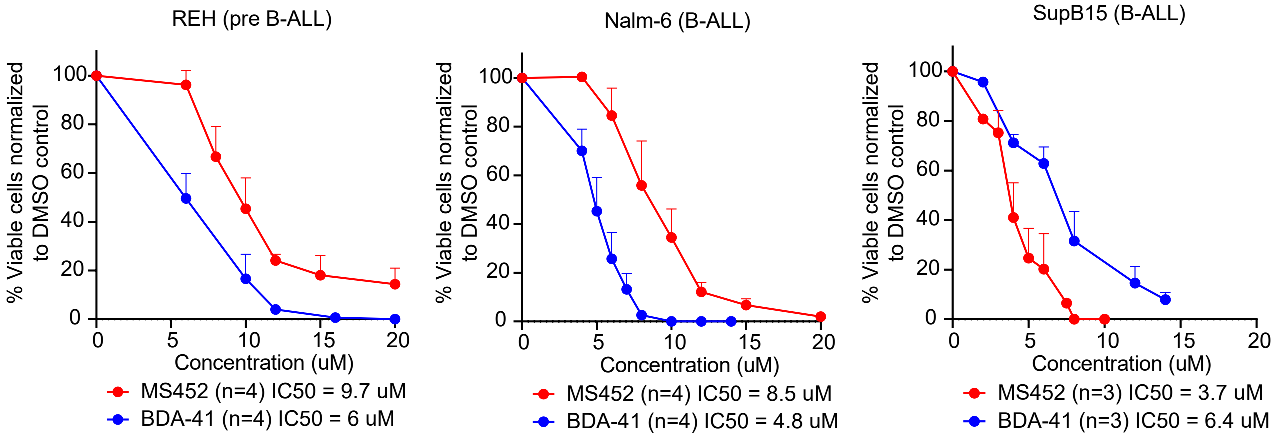

Supplement Figure 4

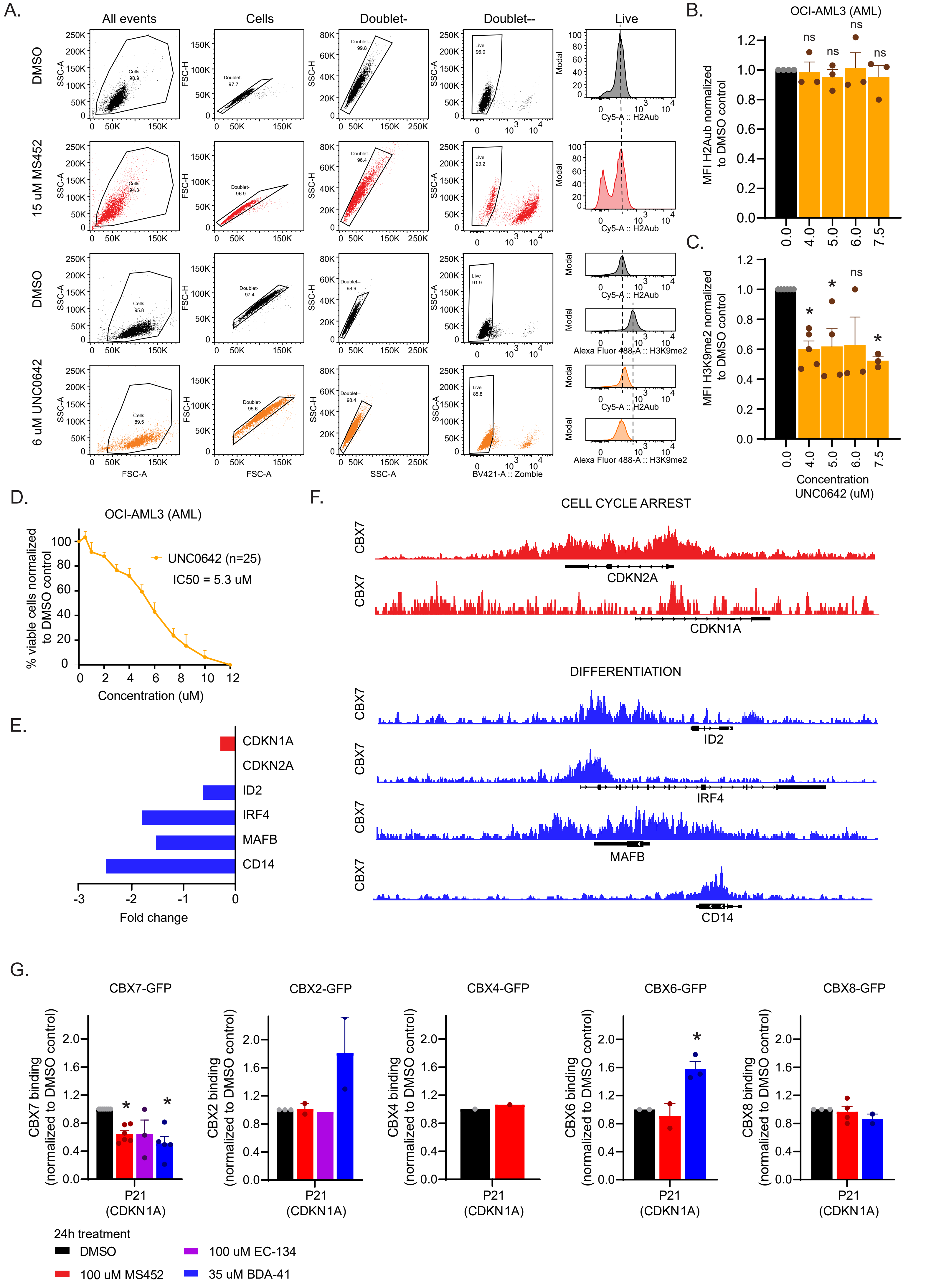

Supplement Figure 5

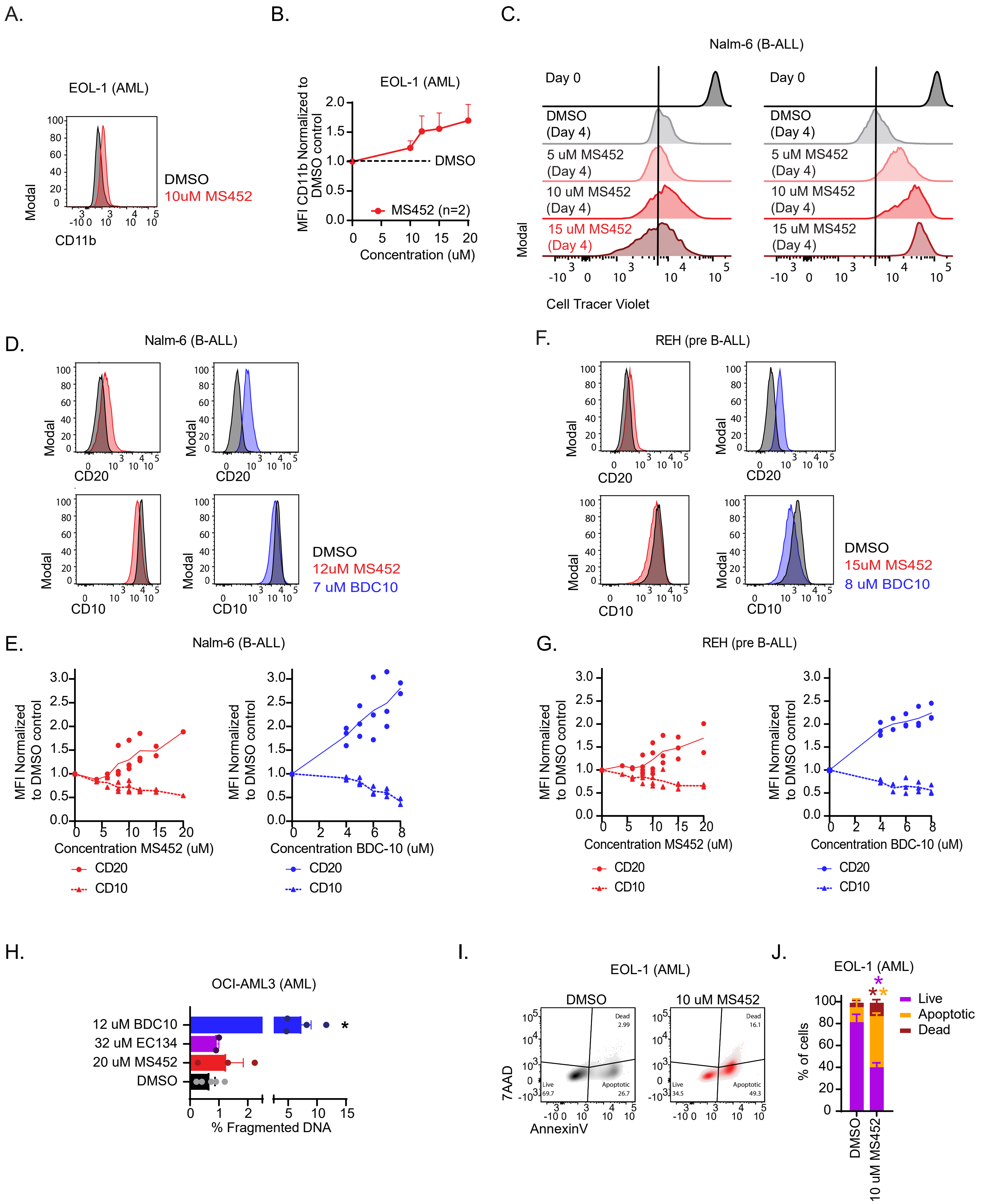

Supplement Figure 6

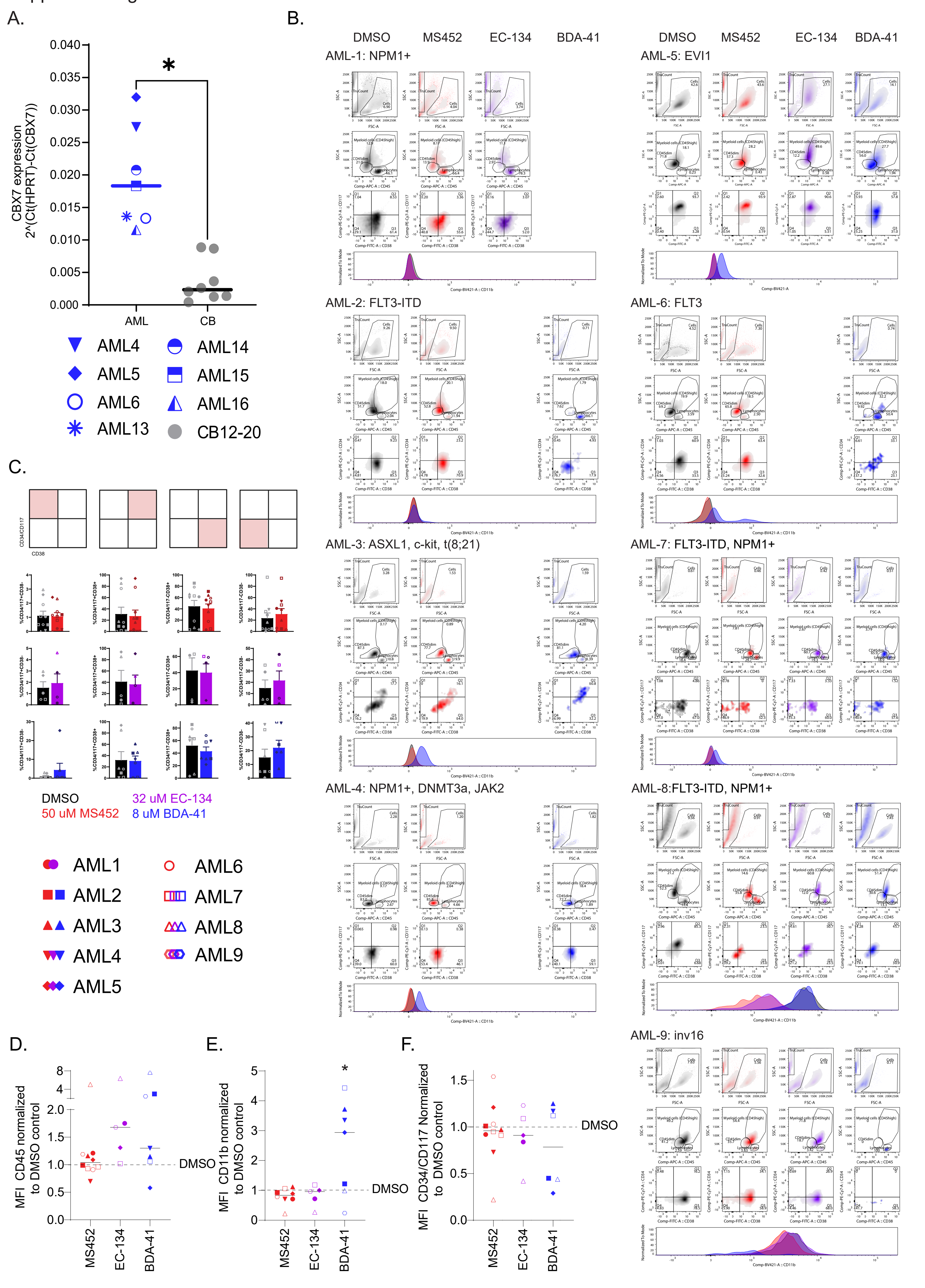

Supplement Figure 6 - continued

G.

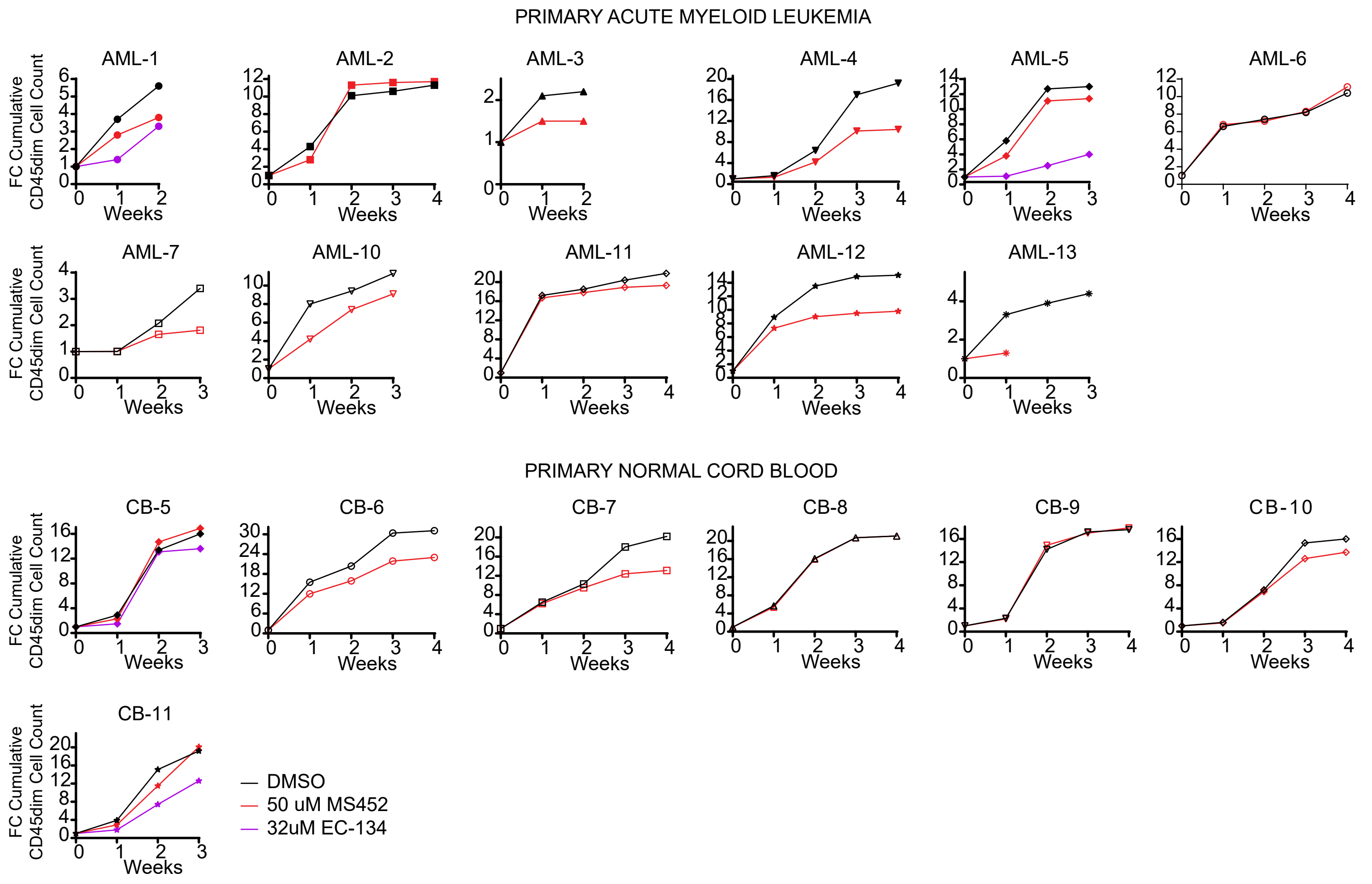

H.

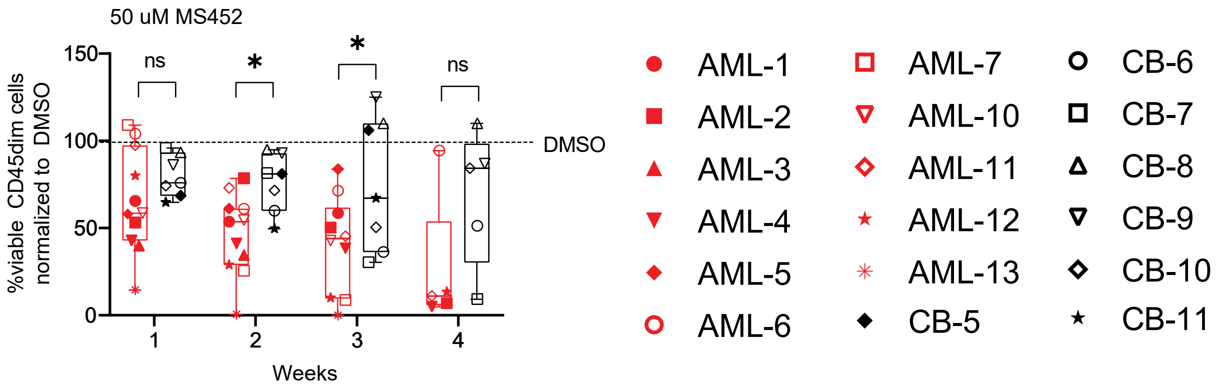

I.

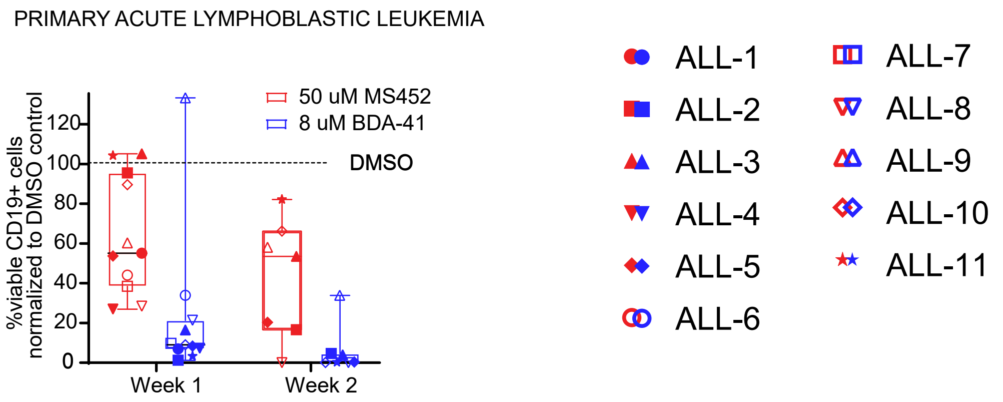

J.

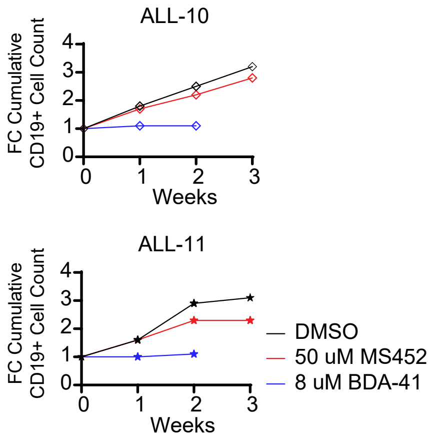

SUPPLEMENTARY TABLES

Table S1. Raw ChIP-qPCR data

Excel file = “Table S1 Raw ChIP-qPCR data”

Table S2. AML and ALL patient information. AML = acute myeloid leukemia, ALL = acute lymphoblastic leukemia, PB = peripheral blood, BM = bone marrow, N/A = not available, N.D. = not detected.

Patient ID	Cell type	Mutation/translocation Status	Material
AML-1	AML	NPM1+	PB
AML-2	AML	FLT3-ITD	BM
AML-3	AML	ASXL1, c-kit, t(8;21)	PB
AML-4	AML	NPM1+, DNMT3a, JAK2	PB
AML-5	AML	EVI-1	BM
AML-6	AML/MDS	FLT3	PB
AML-7	AML	FLT3-ITD, NPM1+	PB
AML-8	AML	FLT3-ITD, NPM1+	PB
AML-9	AML	inv16	PB
AML-10	AML	NPM1+	BM
AML-11	AML	ASXL1, EVI1+	BM
AML-12	AML	ASXL1, RUNX1	PB
AML-13	AML	ASXL1, RUNX1	PB
AML-14	AML	FLT3-ITD, NPM1+, TET2, CEBPA	PB
AML-15	AML	TET2, SF3B1, SRSF2	PB
AML-16	AML	N.D.	PB
ALL-1	B-ALL	N/A	BM
ALL-2	preB-ALL	N/A	BM
ALL-3	ALL	Trisomy-21	BM
ALL-4	B-ALL	N/A	BM
ALL-5	T-ALL	N/A	BM
ALL-6	ALL complex	N/A	BM
ALL-7	N/A	N/A	N/A
ALL-8	N/A	N/A	N/A
ALL-9	N/A	N/A	N/A
ALL-10	preB-ALL	N/A	BM
ALL-11	preB-ALL	N/A	PB

Table S3. Oligonucleotides used throughout the study. List of oligonucleotides used throughout the study.

Type	ID	Sequence ('5 → '3)	Related to figure
guide RNA	crRNA4	TCTTCCTATACCCCGATGCT	1, S1
Primer	genomicCBX7_exon2_Fw	AACTTACCCAAGTGCCCCAC	1, S1
Primer	genomicCBX7_exon2_Rev	GCCCTCCACGTTTGTCAGAT	1, S1
Primer	chipCDKN1A.Fw	ACTGTATGAGGTCAGAACCCCGCC	3, S4
Primer	chipCDKN1A.Rev	CCAGCCAAATAGGTCACTGTGCCG	3, S4
Primer	chipINK4A.Fw	ATCGCTGAGCGATGAAGGTAG	3
Primer	chipINK4A.Rev	ATCACAAAAAGGAAAGGCAAG	3
Primer	chipID2.Fw	GGCTGTCACTAGGAGATCCG	3
Primer	chipID2.Rev	TCACGCACAGCTCAATCTACA	3
Primer	chipIRF4.Fw	AATTCCTCAGGCCACTCAGC	3
Primer	chipIRF4.Rev	TGGATTCGCAGTCCAGTCAC	3
Primer	chipMAFB.Fw	ACGATTAAGCAGATCCCGGC	3
Primer	chipMAFB.Rev	CTTGGGGTCGCACTTTATGC	3
Primer	chipCD14.Fw	GGAGTTCATTGAGCCCTCGT	3
Primer	chipCD14.Rev	CCTGACACTGGACGGGAATC	3
Primer	chipRPL27.Fw	TCCGGACGCAAAGCTGTCATCG	3
Primer	chipRPL27.Rev	TCTTGCCCATGGCAGCTGTCAC	3
Primer	cdnaCDKN1A.Fw	TGAGCCGCGACTGTGATG	3
Primer	cdnaCDKN1A.Rev	GTCTCGGTGACAAAGTCGAAGTT	3
Primer	cdnaINK4A.Fw	CGCTGCCCAACGCACCGAATAG	3
Primer	cdnaINK4A.Rev	ACTCGGGCGCTGCCCATC	3
Primer	cdnaID2.Fw	AGGAAAAACAGCCTGTCGGA	3
Primer	cdnaID2.Rev	GAGCTTGGAGTAGCAGTCGT	3
Primer	cdnaIRF4.Fw	GCTCACGTAGAACCTCTGCT	3
Primer	cdnaIRF4.Rev	CGTAGCCCCTCAGGAAATGT	3
Primer	cdnaMAFB.Fw	CTCAGCACTCCGTGTAGCTC	3
Primer	cdnaMAFB.Rev	GTAGTTGCTCGCCATCCAGT	3
Primer	cdnaCD14.Fw	ACTTGCACTTTCCAGCTTGC	3
Primer	cdnaCD14.Rev	GCCCAGTCCAGGATTGTCAG	3
Primer	cdnaHPRT.Fw	GAACGTCTTGCTCGAGATGTG	3, S6
Primer	cdnaHPRT.Rev	TCCAGCAGGTCAGCAAAGAAT	3, S6
Primer	cdnaCBX7.Fw	GCGGAAGGGTAAAGTCGAGT	S6
Primer	cdnaCBX7.Rev	ACCTCTCTTCCTATACCCCGA	S6

REFERENCES

1. Sanjana NE, Shalem O, Zhang F. Improved vectors and genome-wide libraries for CRISPR screening. Nat Methods. 2014;11(8):783-4.
2. Milosevich N, Wilson CR, Brown TM, Alpsoy A, Wang S, Connelly KE, et al. Polycomb Paralog Chromodomain Inhibitors Active against Both CBX6 and CBX8*. ChemMedChem. 2021;16(19):3027-34.
3. Wang C, Zhang B, Kruger A, Du X, Visser L, Domling ASS, et al. Discovery of Small-Molecule Allosteric Inhibitors of PfATC as Antimalarials. J Am Chem Soc. 2022;144(41):19070-7.
4. Vandamme J, Volkel P, Rosnoblet C, Le Faou P, Angrand PO. Interaction proteomics analysis of polycomb proteins defines distinct PRC1 complexes in mammalian cells. Mol Cell Proteomics. 2011;10(4):M110 002642.
5. Azkanaz M, Rodriguez Lopez A, de Boer B, Huiting W, Angrand PO, Vellenga E, et al. Protein quality control in the nucleolus safeguards recovery of epigenetic regulators after heat shock. Elife. 2019;8.
